# Universal whole-genome Oxford nanopore sequencing of SARS-CoV-2 using tiled amplicons

**DOI:** 10.1038/s41598-023-37588-x

**Published:** 2023-06-26

**Authors:** Ruslan Kalendar, Ulykbek Kairov, Daniyar Karabayev, Akbota Aitkulova, Nuray Tynyshtykbayeva, Asset Daniyarov, Zhenis Otarbay, Saule Rakhimova, Ainur Akilzhanova, Dos Sarbassov

**Affiliations:** 1 Center for Life Sciences, National Laboratory Astana, Nazarbayev University, Astana, Kazakhstan; 2 Institute of Biotechnology, Helsinki Institute of Life Science (HiLIFE), University of Helsinki, Helsinki, Finland; 3 Institute for Molecular Medicine Finland (FIMM), HiLIFE, University of Helsinki, Helsinki, Finland; 4 Astana IT University, Astana, Kazakhstan; 5 School of Sciences and Humanities, Nazarbayev University, Astana, Kazakhstan

**Keywords:** High-throughput screening, Respiratory system models, Next-generation sequencing, Targeted resequencing

## Abstract

We developed a comprehensive multiplexed set of primers adapted for the Oxford Nanopore Rapid Barcoding library kit that allows universal SARS-CoV-2 genome sequencing. This primer set is designed to set up any variants of the primers pool for whole-genome sequencing of SARS-CoV-2 using single- or double-tiled amplicons from 1.2 to 4.8 kb with the Oxford Nanopore. This multiplexed set of primers is also applicable for tasks like targeted SARS-CoV-2 genome sequencing. We proposed here an optimized protocol to synthesize cDNA using Maxima H Minus Reverse Transcriptase with a set of SARS-CoV-2 specific primers, which has high yields of cDNA template for RNA and is capable of long-length cDNA synthesis from a wide range of RNA amounts and quality. The proposed protocol allows whole-genome sequencing of the SARS-CoV-2 virus with tiled amplicons up to 4.8 kb on low-titer virus samples and even where RNA degradation has occurred. This protocol reduces the time and cost from RNA to genome sequence compared to the Midnight multiplex PCR method for SARS-CoV-2 genome sequencing using the Oxford Nanopore.

## Introduction

Severe acute respiratory syndrome coronavirus 2 (SARS-CoV-2) originated in Wuhan, China at the end of December 2019 and has spread rapidly around the world. By 17 February 2023, infections with SARS-CoV-2 have resulted in more than 6.8 million deaths (https://covid19.who.int/). Given the current situation in the world and our country in connection with the COVID-19 pandemic, the entire medical community and the Government of the Republic of Kazakhstan are developing measures against the spread of the virus in the country^1^. Globally, research has been initiated on the genetics of SARS-CoV-2, human genetics, and the relationship between the course and severity of SARS-CoV-2 infection from various external and internal (human body) risk factors^2^. Analysis of the genomic data of pathogen variants is paramount in the study of the epidemiological processes of infectious diseases. Genetic sequence data elucidate key epidemiological parameters, such as the reconstruction of transmission routes, time of origin, and identification of possible sources and reservoirs of infection. Ideally, drugs and vaccines should target invariant regions of the genome to prevent drug resistance and ensure vaccine efficacy^2,3^. Therefore, continuous monitoring of genomic changes in the virus is essential to better understand the fundamental interactions between the host and pathogen. Large-scale genome-wide sequencing of emerging and constantly circulating viral pathogens provides critical information about viral evolution and supports epidemiological investigations, as recently demonstrated in the Ebola and Zika virus outbreaks^4–8^. At present, constant monitoring of genomic changes in SARS-CoV-2 indicates the formation of phylogenetic clades, with geographical clustering. Homoplasy mutations have also been identified that appear in different regions, which indicate an ongoing adaptation of the virus to the new, human host, during which the virus can increase or decrease its virulence^9^. Better understanding of genomic changes is possible only by sequencing viral genomes directly from clinical samples, which is a faster, more productive, and less laborious method than the use of preliminary isolation of viruses in cell culture. To increase the number of genomic copies of viral cDNA, an enrichment using multiplex PCR is often used with numerous primer pairs in one reaction. The result is a set of short DNA fragments that overlap across the entire genome of the virus. Targeted enrichment based on PCR is a key step, as different target regions can be freely combined or excluded through primer selection. The main difficulty of enrichment-based PCR is to ensure representative amplification of all regions of the virus genome, and subsequent uniform coverage during sequencing using a high-performance next-generation sequencing (NGS) platform. This requires careful design of compatible PCR primers with similar thermodynamic parameters, such as melting points and Gibb’s free energy. Combining several primers in the same reaction will create the likelihood of unwanted interactions between these primers, which negatively affect the amplification itself and therefore sequence coverage. Thus, an important step for optimization is the allocation of primers to different pre-amplification and multiplex PCR reactions to avoid unwanted primer interactions. In this regard, the development of a universal platform for targeted amplification and sequencing of the SARS-CoV-2 genome is one of the most critical tasks. The recently developed Oxford Nanopore Technologies (ONT) allows single-molecule sequencing of long-size DNA fragments. Commercial panels for PCR-based targeted enrichment of the SARS-CoV-2 genome sequencing are currently available. The ARTIC SARS-CoV-2 workflow for library preparation method (Midnight protocol) employs a 1.2–2.0 kb PCR amplicon tiling approach to ensure an even depth of coverage across the full RNA virus genome, even where RNA degradation has occurred^10,11^. Further, the main disadvantage is that genome coverage is lost for regions that fail to amplify in the multiplex PCR^10,12,13^. Here, we developed a comprehensive multiplexed set of primers adapted for the Oxford Nanopore rapid barcode library kit that allows universal SARS-CoV-2 genome sequencing. This primer set is designed to set up any variants of primer pools for whole-genome sequencing of SARS-CoV-2 using single- or double-tiled amplicons from 1.2 kb up to 4.8 kb. The proposed protocol allows whole-genome sequencing of SARS-CoV-2 with tiled amplicons up to 4.8 kb on low-titer virus samples and even where RNA degradation has occurred.

## Materials and methods

### Clinical RNA SARS-CoV-2 isolate

For surveillance studies, a set of residual nasopharyngeal swab specimens positive for SARS-CoV-2 qRT-PCR from the Republican Diagnostic Centre (RDC) (https://umc.org.kz/en/) and private laboratory KDL “Olymp” (https://www.kdlolymp.kz/) were collected across the Republic of Kazakhstan between 2020 and 2022. 341 sequences that passed quality control are deposited in the Global initiative on sharing all influenza data (GISAID) (https://www.gisaid.org/hcov19-variants/) under the accession number: [EPI_ISL_13717748-EPI_ISL_13717798; EPI_ISL_13717799-EPI_ISL_13718011; EPI_ISL_5532919-EPI_ISL_5532925] and can be found in online repositories (National Center for Biotechnology Information Sequence Read Archive under accession number PRJNA915200). The clinical isolate total RNA with SARS-CoV-2 was stored at − 80 °C. Viral RNA was isolated from clinical biomaterials using ALPREP extraction kit following the manufacturer’s (Algimed Techno, Belarus) instructions at the RDC laboratory. To determine Ct values, we additionally performed quantitative RT-PCR using ALSENSE-SARS-CoV-2-qPCR kit (Algimed Techno, Belarus) following the manufacturer’s instructions. Ct < 32 was applied for sequencing for SARS-CoV-2 qRT-PCR positive sample selection. We used a 16-µl mix of reaction buffer and reverse transcriptase according to the manufacturer’s specifications using a 10-µl RNA template. Reactions were run at a total volume of 26 µl on the real-time PCR device. All methods were performed in accordance with the relevant guidelines and regulations. The sampling protocol was approved by the ethics committee of Corporate Fund “University Medical centre”.

### Design of multiplex primer sets

Two pools of a total of 48 primer pairs (Supplementary Table S1) were designed using FastPCR software (http://primerdigital.com/fastpcr.html) to cover the whole SARS-CoV-2 genome^14,15^, based on the consensus SARS-CoV-2 sequence genome obtained from the multiple alignments of complete SARS-CoV-2 genomes available in NCBI and GISAID from the beginning of the outbreak (until August 2021). Each primer pair covers approximately 1.2 kb of the genome with about 700-bp overlap of amplicons (Supplementary Table S1). The following FastPCR software setting for designing multiplex tiling PCR was used to develop two overlapping PCR pools for tiling fragments of approximately 1.2 kb: -tiling[-700] (1150–1250) -ln21-28 -tm55-58 -3tm23-32 -q70 -lc70 -nomxbpr. The development of PCR-compatible primer pairs was performed with the expectation that primers from different pools could be applied in multiplex PCR. That is, it was possible to build any sets of compatible primer pairs for any tasks related to detection and sequencing on any platform, including for quantitative and qualitative analysis of any SARS-CoV-2 sequence genome sites. Therefore, we originally developed multiplex tiling PCR pools of 1.2 kb that can be adapted to any other size from 1.2 to 4.8 kb or longer, including primers from different pools (Supplementary Tables S1–S4).

### cDNA generation

The clinical isolate total RNA with SARS-CoV-2 was directly used for first-strand synthesis using the Maxima H Minus Reverse Transcriptase (Thermo Fisher Scientific) with a set of SARS-CoV-2 specific primers (24 primers, from the only reverse direction from pool B: B2-B48) (Supplementary Table S5). The 24 primers used for cDNA synthesis from the reverse direction were located approximately 1.2 kb apart for the most efficient coverage and full-genome synthesis of the SARS-CoV-2 genome cDNA. In the case that certain primers would not be efficient enough for synthesis (such as situations in which SARS-CoV-2 sequences have insertion-deletions or mismatches), the neighboring primers could substitute them with long cDNA fragments synthesis. The Maxima H Minus Reverse Transcriptase allows efficient synthesis of very long cDNA fragments (up to 20 kb) at very low viral RNA concentrations.

Each RNA sample was used for multiple cDNA reactions. All reactions were performed in 0.2 ml 96-well plates. cDNA synthesis reactions were prepared with 2 × RT mix for analysis of 96 RNA samples (Table 1). The 2 × RT mix was calculated for 100 samples in 2 × ratio at a 500-µl volume: 200 µl 5 × Reverse Transcriptase buffer (375 mM KCl, 15 mM MgCl_2_, 50 mM DTT, 250 mM Tris–HCl, pH 8.3 at 25 °C), 50 µl 10 mM each dNTP (1 mM final concentration), 10 µl 1 µM each primer mix (24 primers, final concentration of each primer 20 nM), 5 µl Maxima H Minus Reverse Transcriptase (200 U/µl) (final concentration 2 U/µl), and 235 µl sterile Milli-Q water. Each RNA sample was added to an equal volume of 2 × RT mix (5 µl). The 96-well plate was placed in a thermocycler and incubated for 1 min at 65 °C followed by 30 min at 50 °C and 5 min at 85 °C. cDNA reactions were stored at − 20 °C.

**Table 1:** Composition of the reaction mixture for cDNA synthesis.

Components	Concentration	Volume (µl)	Final concentration
Reverse Transcriptase buffer (375 mM KCl, 15 mM MgCl_2_, 50 mM DTT, 250 mM Tris–HCl, pH 8.3 at 25 °C)	5 ×	2	1
dNTP	10 mM	0.5	0.5 mM
Primer mix (24 even primers, from only the reverse direction from the pool B: B2-B48 (Table S5)	1 µM of each primer	0.1	10 nM
Maxima H Minus Reverse Transcriptase	200 U/µl	0.05	1 U/µl
Fresh Milli-Q water (Biopak Polisher)		2.35	
Master mix		5	
RNA template	NA	5	
Total		10	

### Primer validation and SARS-CoV-2 sequence genome quality analysis

To test the efficiency of each primer pair, we performed conventional PCR with Phusion Hot Start II DNA Polymerase (Thermo Fisher Scientific). During primer pair validation, we also examined the quantitative efficiency of amplification of different SARS-CoV-2 sequence genome sites for intact and degraded SARS-CoV-2 sequence. The efficiency of amplification of fragments from the 3'- and 5'- SARS-CoV-2 sequence genome was used to determine the integrity of the virus sequence and the level of degradation.

**Figure 1:**
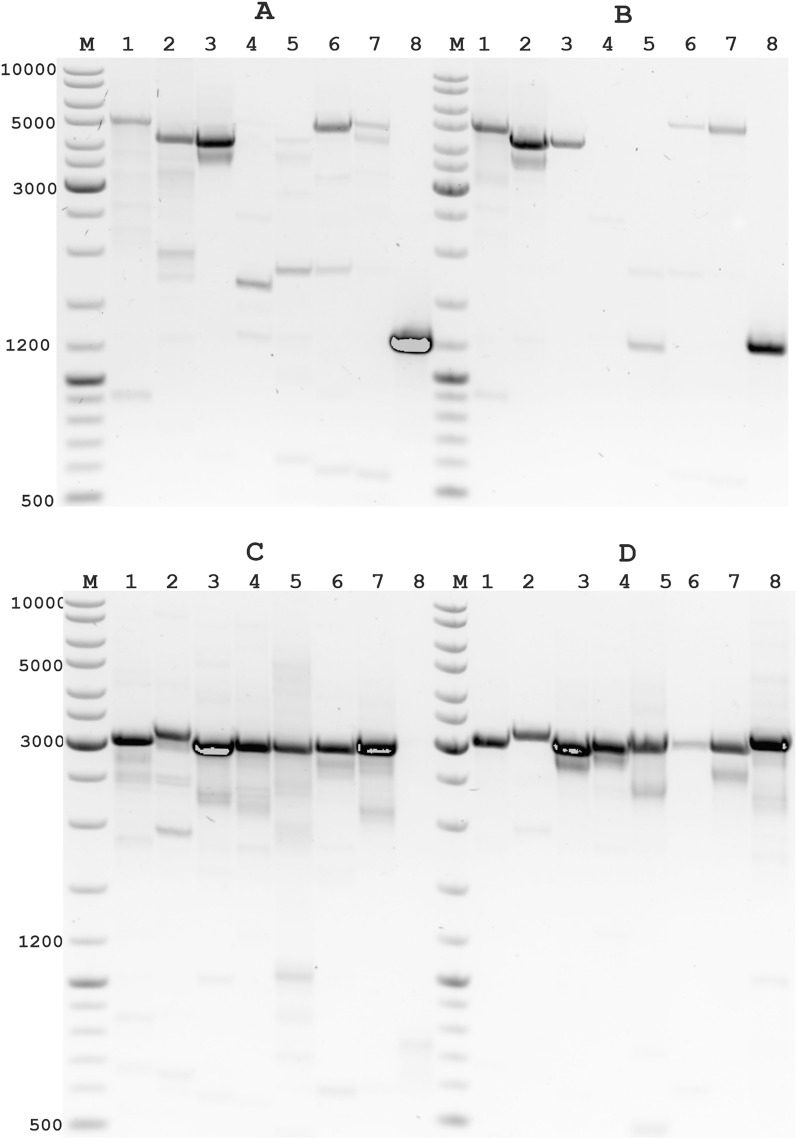
Efficacy of long-distance PCR for cDNA of SARS-CoV-2 obtained with a proposed protocol based on Maxima H Minus reverse transcriptase and SARS-CoV-2 specific primers. A and B, results of long-fragment amplification around 4800 bp; C and D, results of fragment amplification around 3000 bp. A and C, cDNA synthesis was performed with specific primers at a final concentration of 20 nM; B and D, - cDNA synthesis was performed with specific primers at a final concentration of 10 nM, respectively. A-B primer pair: 1. B48-B41 (4873 bp); 2. B48-A43 (4255 bp); 3. B44-A39 (4167 bp); 4. B40-B33 (4764 bp); 5. B40-A35 (4139 bp); 6. A25-A32 (4795 bp); 7. A29-A36 (4775 bp); 8. B43-B44 (1200 bp). C-D primer pair: 1. A17-B20 (3012 bp); 2. A21-B24 (3113 bp); 3. A25-B28 (2928 bp); 4. A29-B32 (2949 bp); 5. A33-B36 (2953 bp); 6. A37-B40 (2936 bp); 7. A41-B44 (2942 bp); 8. A45-B48 (3041 bp). M-Thermo Scientific GeneRuler DNA Ladder Mix.

Multiplex PCR validation was used for this task with two pairs A05-A08 (2.4 kb) and A45-B48 (3.041 kb). For analysis, 96 samples of the reaction mix were calculated for a total volume of 1000 µl: 755 µl Milli-Q water, 200 µl 5 × Phusion HF Buffer (with 7.5 mM MgCl_2_), 20 µl dNTP (10 mM), 10 µl Phusion Hot Start II DNA Polymerase (2 U/µl), 8 µl 5 µM of each primer (A05-A08), and 8 µl 5 µM of each primer (A45-B48) (final concentration of each primer 40 nM). The reaction mix was transferred (10 µl) to PCR plate wells and 1 µl cDNA sample was added. PCR amplification was carried out under the following conditions: initial denaturation step at 98 °C for 30 s, 34 amplification cycles at 98 °C for 10 s, 68 °C for 60 s, and 72 °C for 60 s. The amplified long PCR products were visualized using 1.2% agarose gel electrophoresis (Fig. 1).

### Multiplex PCR for A and B pools

A multiplex PCR protocol for each pool using low primer concentrations and high primer annealing temperatures (65–72 °C) and extended time for annealing was used. This protocol allows equal amplification of each product covering the whole virus genome. We designed two primer pools such that neighboring amplicons were adjacent to or at some distance from within the same pool. By screening reaction conditions based on the multiplex amplification and specificity as determined by gel electrophoresis, we identified the primer concentrations and PCR conditions.

Two separate multiplexed PCR reactions (Pools A and B) were generated using the Phusion Hot Start II DNA Polymerase and the primer scheme described in Supplementary Table S2. These two pools of primers contain 12 primer pairs each that generate the odd- or even-numbered tiled amplicons for the 2.4 kb set. Primer pools were prepared where each primer was pre-pooled to 400 nM. For analysis, 96 samples of reaction mix were calculated for a volume of 1800 µl: 1190 µl Milli-Q water, 400 µl 5 × Phusion HF Buffer (with 7.5 mM MgCl_2_), 40 µl dNTP (10 mM), 20 µl Phusion Hot Start II DNA Polymerase (2 U/µl), and 150 µl 400 nM primer pool A/B (final concentration of each oligo 30 nM). The reaction mix (18 µl) was transferred to a PCR plate and 2 µl of cDNA was added (Table 2). PCR amplification was performed under the following conditions: initial denaturation step at 98 °C for 30 sec, 34 amplification cycles at 98 °C for 10 sec, and 65 °C for 5 min.

**Table 2:** Composition of the reaction mixture for multiplex PCR.

Components	Concentration	Volume (µl)	Final concentration
Phusion HF Buffer (with 7.5 mM MgCl_2_)	5 ×	4	1 ×
dNTP	10 mM	0.4	0.2 mM
Primer pools A/B	400 nM each primer	1.5	30 nM
Phusion Hot Start II DNA Polymerase	2 U/µl	0.2	0.02 U/µl
Fresh Milli-Q water (Biopak Polisher)		11.9	
Master mix		18	
cDNA template	NA	2	
Total		20	

The PCR products in pool A and B reactions for the same samples were combined and purified with 1 × AMPure XP and quantified with Qubit Broad Range Kit on SpectraMax (Molecular Devices, San Jose, CA).

### ONT library preparation and sequencing

The library was prepared using the Midnight RT PCR Expansion (EXP-MRT001). Two separate PCR reactions with pool A and pool B primer sets for each sample were pooled, followed by rapid barcoding using the Rapid Barcoding kit (SQK-RBK110.96, Oxford Nanopore Technologies) and SPRI bead DNA clean-up, which were performed according to the manufacturers’ instructions. Samples were run in duplicate, and a negative control was used in each round for experimental validation. Up to 96 samples were pooled for sequencing in one flow cell^11,16^.

### Whole genome sequencing with Oxford nanopore technologies

A GridION/PromethION run using the FLO-MIN106D flow cell version R9.4.1 was performed consisting of 95 positive samples and 1 negative control (present in all steps post RNA extraction). Sequencing was performed for 12 to 24 h using the high accuracy base calling in the MinKNOW software. High-accuracy base calling was performed after sequencing from the fast5 files using the Oxford Nanopore Guppy tool. The run was monitored by RAMPART (https://github.com/articnetwork/rampart) such that it could be stopped when ≥ 20 × depth for all amplicons was achieved    (SupplementalData file).

### Software setup and installation

The ARTIC sequencing data obtained by Midnight protocol was analyzed by the wf-artic bioinformatics pipeline. The Nextflow software orchestrates this workflow. The Nextflow and Docker software are required for wf-artic pipeline installation. The NextFlow project is available in open access and allows the implementation of a scientific pipeline in a reproducible manner. The software installation on the Linux operating system is straightforward and is supported on GridION and PromethION devices. After the demultiplexing step, the sequence reads were processed by ARTIC FieldBioinformatics software that was adapted to analyze FASTQ Nanopore sequences. The ARTIC pipeline was also modified to utilize a primer scheme that specifies the sequencing primers used in the Midnight protocol and their genomic localization on the SARS-CoV-2 genome. The wf-artic pipeline classifies the sequenced samples according to Nexclade clastidic analysis and Pangolin strain assignment.

### Demultiplexing of multiple barcoded samples

Demultiplexed FASTQ format sequence data is required for the wf-artic workflow (https://github.com/epi2me-labs/wf-artic). Guppy v6.1.5 performs basecalling of all reads and identifies barcodes in the sequence. Guppy barcoding performs basecalling of all reads and identifies barcodes in the sequence. To prevent re-basecalling, the software copies the reads about each barcode to the corresponding tag output directory. Since Midnight protocol utilizes a rapid barcoding kit, the demultiplexing step does not need barcodes at both ends of the sequence. In addition, filtering against mid-strand barcodes is not required.

### Variant calling and phylogenetic profiling

Sequencing reads aligned by using minimap2 v2.18^17^. Medaka v1.5 (https://github.com/nanoporetech/medaka) is used for the generation of consensus sequences from basecalled data. Genetic variants were identified using bcftools v1.12^18^. Finally, wf-artic pipeline generated several outputs (html, vcf and fasta) and includes results of NextClade^19^ and Pangolin analysis with the clade designation according to Pangolin nomenclature. Multiple sequence alignment (MSA) using the MAFFT software^20^ and IQ-TREE2 tool^21^ were implemented to construct a phylogenetic tree for our SARS-CoV-2 samples from two primer sets (Set 1-Nanopore protocol, Set 2-Our custom protocol). FigTree tool (http://tree.bio.ed.ac.uk/software/figtree/) used for visualization of generated the phylogenetic tree.

### Ethical approval

The sampling protocol was approved by the ethics committee of Corporate Fund “University Medical centre” (#20/2020, 16th November 2020). The genomic study was approved by the research ethics committee of the PI “National Laboratory Astana” (#01-2022 from 2nd March 2022). Institutional written informed consent about nationality declaration, RNA extraction and further investigation was signed and obtained from the participating individuals.

## Results

### Tiled primers design

The tiled amplicon set design resulted in uniform coverage of the SARS-CoV-2 consensus genome sequence, resulting in high-quality, complete genomes with minimal gaps in sequencing. Each primer pair covered approximately 1.2 kb of the genome with about 700 bp overlap of amplicons (Supplementary Table S1). The primer binding position was in the most conserved regions of the SARS-CoV-2 genome, and primer sequences did not contain degenerate nucleotides. We designed the tiled amplicon set for two pools (Pool A and Pool B) such that the overlapping amplicon from the Pool B set begins to the left of the middle of the previous amplicon from Pool A. This careful coverage allowed repeat sequencing of the same sites, in case of failure for primers during the accumulation of mutations in the SARS-CoV-2 genome sequence. Therefore, the development of PCR-compatible primer pairs was performed with the expectation that primers from different pools could be applied at multiplex PCR. That is, it was possible to build any sets of compatible primer pairs for any tasks related to detection and sequencing on any platform, including for quantitative and qualitative analysis of any SARS-CoV-2 sequence genome sites. We developed multiplex tiling PCR pools of 1.2 kb, which can be adapted to any other size from 1.2 to 4.8 kb or longer, including primers from different pairs (Fig. 1). The developed tiled amplicon set for two pools can be easily designed for specific tasks, both for the detection of highly degraded viral sequences and for different sequencing technologies targeting specific sequencing lengths using Third Generation Sequencing (TGS) technologies.

### Protocol improvements, cDNA generation, and SARS-CoV-2 sequence genome quality analysis

The library preparation protocol was improved at the stage of cDNA synthesis and multiplex PCR. We addressed several limitations of Midnight cDNA synthesis and multiplex PCR to eliminate systematic dropouts and increase the sensitivity sequencing on low-titer virus samples. As a versatile platform, this protocol is primarily intended for a variety of tasks, including targeting specific genes or viral RNA detection, and for rapid and inexpensive SARS-CoV-2 whole-genome sequencing adapted to existing NGS or TGS technologies. Since our initial goal was high-sensitivity whole-genome sequencing based on ONT technology, we focused on generating a long-tiled amplicon. In addition, the use of a set of SARS-CoV-2 specific primers at the cDNA synthesis stage allowed us to significantly increase the sensitivity of virus genome detection, including for samples with very low concentrations and with degraded viral RNA. Since the efficiency of the Maxima H Minus Reverse Transcriptase allows efficient synthesis of very long cDNA fragments (up to 20 kb) at very low viral RNA concentrations, the potential of our protocol can be applied for sequencing fragments longer than 5 kb. The SARS-CoV-2 specific primer more efficiently produces cDNA and a fragment size that can achieve the maximum polymerase processivity for commercial reverse transcriptase, which significantly increases both the sensitivity of PCR detection and guarantees SARS-CoV-2 whole genome sequencing, including for new and unknown variants of the virus. According to the manufacturer’s recommendations for using long RT-PCR (> 5 kb), we used a final concentration of 1 U of Maxima H Minus Reverse per 1 µl reaction mixture and incubation time (50 °C for 30 min). We used these reaction conditions, among others, for cDNA synthesis for very degraded RNA with low viral RNA concentration. In this way, we optimized the protocol and reaction conditions at the initial stage and library preparation. Further steps were based on using the Oxford Nanopore Rapid Barcoding kit and a bead-based clean-up step after PCR according to the ONT protocol.

**Figure 2:**
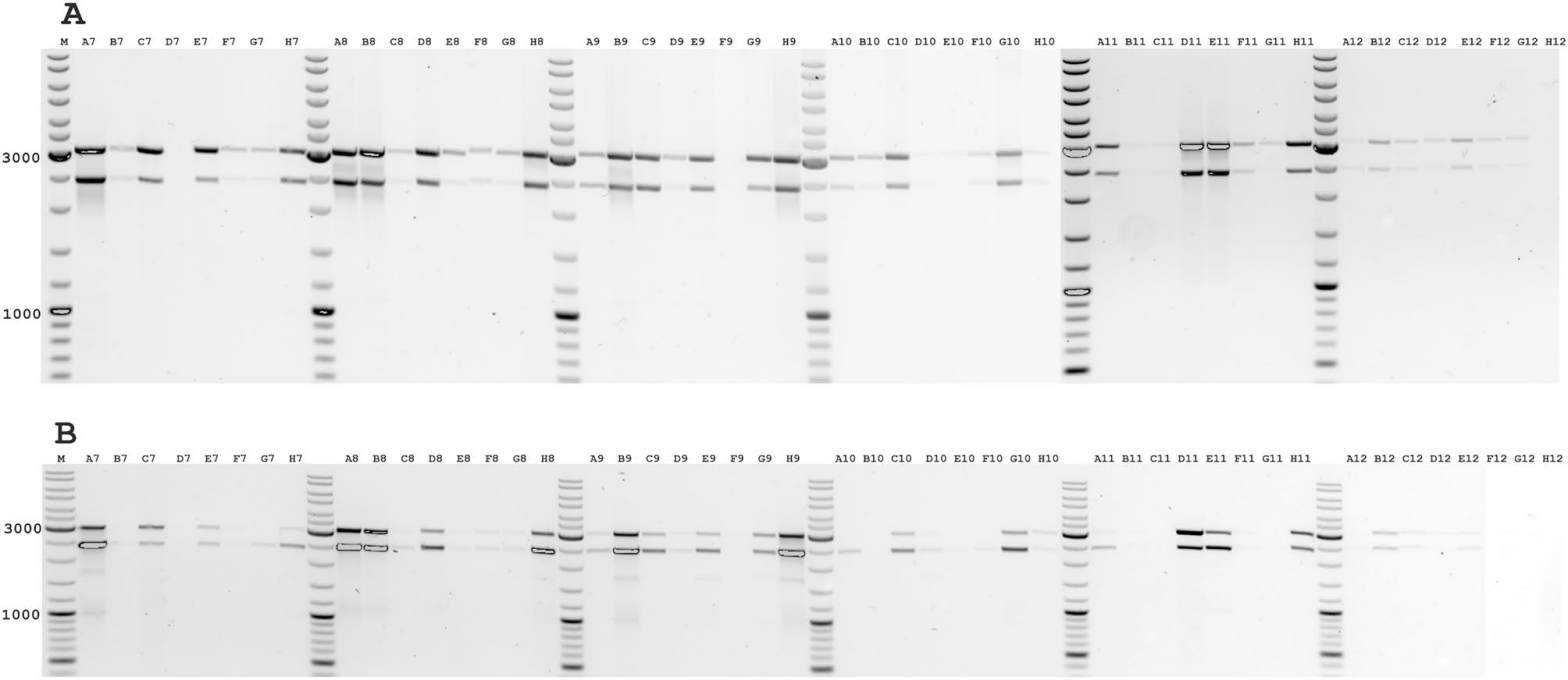
Comparative analysis for SARS-CoV-2 positive samples by multiplex PCR with B48-A45 (3041 bp) and A05-A08 (2400 bp) pairs. The same 96 samples were analyzed in parallel; the name of the samples corresponds to the numbers on the 96-well plate (H12 was control sample without RNA). A, cDNA synthesis was performed with SARS-CoV-2 specific primers and Maxima H Minus Reverse Transcriptase. B, cDNA synthesis was performed with random primers and LunaScript Reverse Transcriptase (EXP-MRT001). M-Thermo Scientific GeneRuler DNA Ladder Mix.

The efficiency of cDNA synthesis using our RT protocol using Maxima H Minus Reverse Transcriptase (Thermo Fisher Scientific) with a set of SARS-CoV-2 specific primers was studied in comparison with a protocol based on LunaScript RT super mix using random primers from Midnight RT PCR Expansion (Oxford Nanopore Technologies). Because the same clinical RNA of SARS-CoV-2 isolates with different concentrations of RNA were used, the efficiency and sensitivity of cDNA synthesis for these RT protocols could be determined (Fig. 2). We performed quantitative PCR to evaluate the efficiency of cDNA synthesis using our RT protocol using Maxima H Minus reverse transcriptase with a set of SARS-CoV-2-specific primers compared with the LunaScript supermix-based RT protocol using random primers. The differences in Ct values we observed for these protocols were at least 2 PCR cycles lower for our RT protocol. In some cases, the differences were greater than 7 PCR cycles or no amplification was detected for cDNA samples obtained using the LunaScript RT-based protocol (Supplementary Table S6).

Multiplex PCR including primer sets containing two primer pairs each for different genome regions of the 3'- and 5'-sequence of SARS-CoV-2 was used for the analysis. The identity of the amplification of these fragments was used to determine the integrity of the virus sequence and the level of degradation. According to our results, the sensitivity and efficiency of our protocol using Maxima H Minus Reverse Transcriptase (Thermo Fisher Scientific) with a set of SARS-CoV-2-specific primers were significantly higher than the protocol based on LunaScript RT super mix using random primers. As many samples with very low concentrations of viral RNA were efficiently amplified using our protocol, we recommend this protocol not only for detection of SARS-CoV-2 RNA but also for efficient detection of other RNA-containing viruses. In addition, we optimized cDNA synthesis conditions for different concentrations of SARS-CoV-2 specific primers. We used a low concentration of each SARS-CoV-2 specific primer in the reaction RT mix, as 5 nM of each SARS-CoV-2 specific primer was already sufficient for efficient cDNA synthesis. However, we observed the highest sensitivity and efficiency of cDNA synthesis from 10 to 20 nM of the final concentration of SARS-CoV-2 specific primer (Fig. 2). For the efficient synthesis of long cDNA fragments, 10 nM of SARS-CoV-2 specific primer is sufficient, even for low-titer virus samples and even where RNA degradation has occurred. In addition, an excessive concentration of specific primer would interfere with the next step (multiplex PCR), as short-tiled amplicons will be formed.

### Verification of the intact virus genome

**Figure 3:**
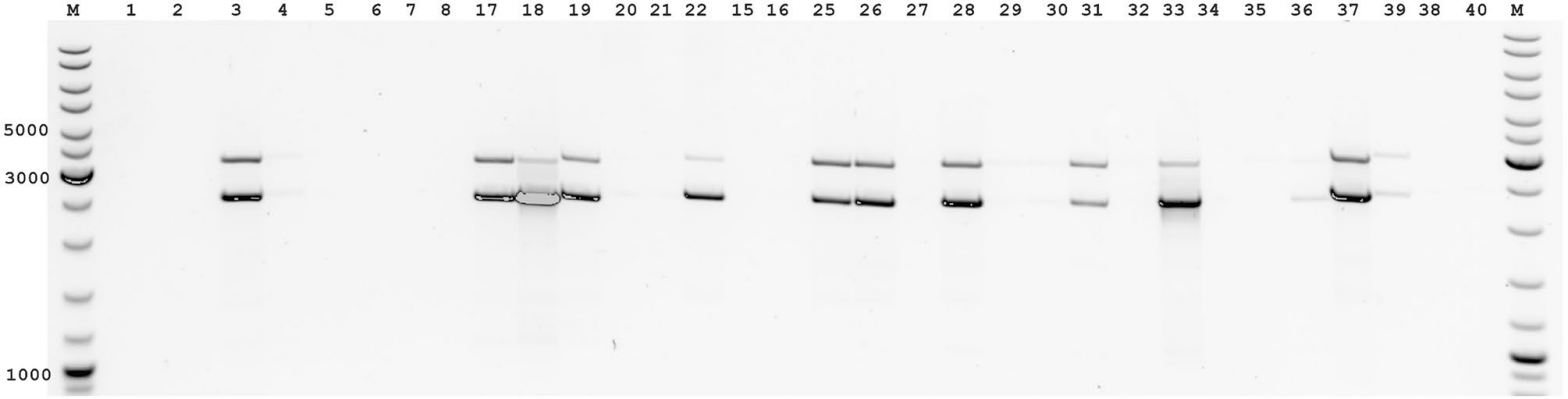
Individual screening of SARS-CoV-2 positive samples with low-titers and where RNA degradation had occurred (stored for about a year in a − 20 °C freezer with multiple cycles of thawing and refreezing) by multiplex PCR with B48-A45 (3041 bp) and A05-A08 (2400 bp) pairs. The A05-A08 primer pair for the amplification of the 2400-bp fragment characterizes the intact region of the 5' sequence of the SARS-CoV-2 genome. The second B48-A45 pair for the amplification of the 3041-bp fragment characterizes the most distant region from the 5' sequence of SARS-CoV-2 genome, located near the poly-A site. M-Thermo Scientific GeneRuler DNA Ladder Mix.

RNA degradation occurs for several reasons, including improper storage. Other issues have exacerbated this problem during this pandemic, such as shortages of lysis buffer and insufficient ultra-low freezer capacity. We examined the quantitative amplification efficiency of different SARS-CoV-2 sequence genome sites for intact and degraded sequence from SARS-CoV-2. The identity of the amplification of these fragments was used to determine the integrity of the virus sequence and the level of degradation. We analyzed the efficiency of amplification of long fragments for relatively old virus samples with low titters and even where RNA degradation had occurred (samples stored for about a year in a -20 °C freezer with multiple cycles of thawing and refreezing). This analysis was performed by multiplex PCR for long fragments with A05-A08 (2400 bp) and B48-A45 (3041 bp) pairs (Fig. 3). Since the primer pairs used correspond to opposite regions of the virus genome, we thus analyzed the integrity of the viral genome. The intact viral RNA sequence was characterized by almost identical intensities of both amplicons for the A05-A08 (2400 bp) and B48-A45 (3041 bp) pairs. The A05-A08 primer pair for the amplification of the 2400-bp fragment characterizes the intact region of the 5'-sequence of SARS-CoV-2 genome. The second B48-A45 pair for the amplification of the 3041 bp fragment characterizes the most distant region from the 5'-sequence of SARS-CoV-2 genome, located next to the poly-A site. In the situation where the amplification intensity of both amplicons for the A05-A08 (2400 bp) and B48-A45 (3041 bp) pairs changes relative to each other, this can provide some insight into the degree of degradation of the viral genome on one side or the other. Thus, for viral genome degradation at the initial site, the efficiency of short-fragment amplification for the A05-A08 pair (2400 bp) will be reduced. We observed a relative decrease in amplification of the longer B48-A45 (3041 bp) amplicon more frequently, which is characteristic of partial degradation of the most distal part of the genome regions of the 3' sequence of SARS-CoV-2 (Fig. 3). Even though these RNA samples contained a little amount and degraded viral RNA, we were able to obtain amplification of long fragments as small as 4.8 kb for these samples (Fig. 3).

### Results comparison with Oxford Nanopore sequencing

**Figure 4:**
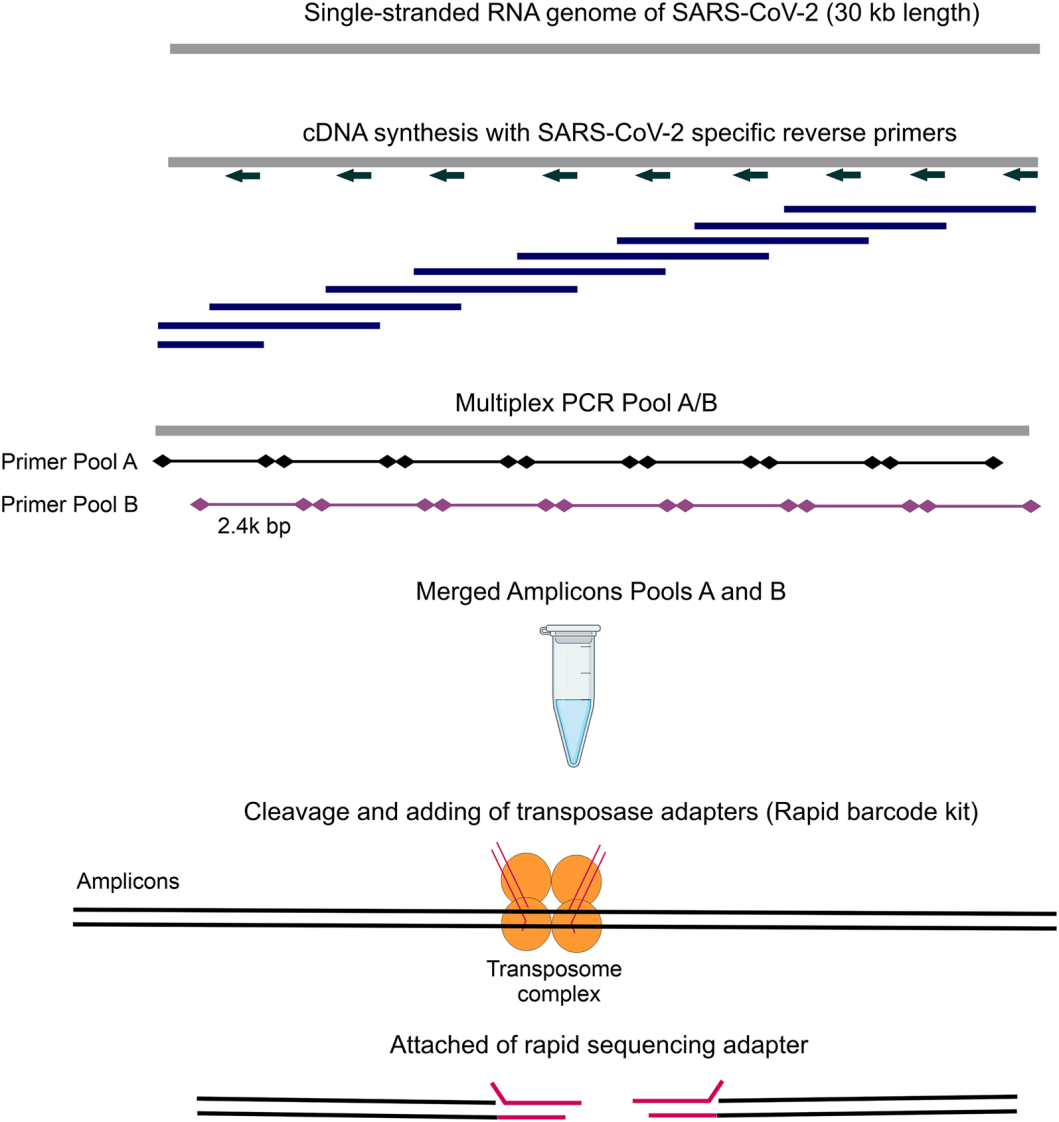
SARS-CoV-2 ONT sequencing workflow, from the RNA sample to loading to sequence analysis. A schematic illustration of the tiling PCR strategy for the whole-genome sequencing of SARS-CoV-2 is shown. First-strand cDNA synthesis using the Maxima H Minus Reverse Transcriptase with a set of SARS-CoV-2 specific primers (24 primers, from the only reverse direction from the pool B: B2-B48) was located approximately 1.2 kb apart. Two separate multiplexed PCR reactions (Pools A and B) were generated using the Phusion Hot Start II DNA Polymerase. These two pools of primers contain 12 primer pairs each that generate the odd- or even-numbered tiled amplicons for the 2.4 kb set with about 550 bp overlap of amplicons. The PCR products in pool A and B reactions for the same samples were combined and purified with AMPure XP and quantified with Qubit Broad Range Kit on SpectraMax. Followed by rapid barcoding using the Rapid Barcoding kit and SPRI bead DNA clean-up. The Oxford Nanopore Rapid Barcoding library kit generates barcoded sequencing libraries from pooled amplicons, the transposase approach simultaneously cleaves amplicons and attaches barcoded tags to the cleaved ends. These amplicons are then subjected to Oxford Nanopore library preparation, using methods that directly add adapters to the amplicons. Samples were pooled for sequencing in one flow cell.

**Figure 5:**
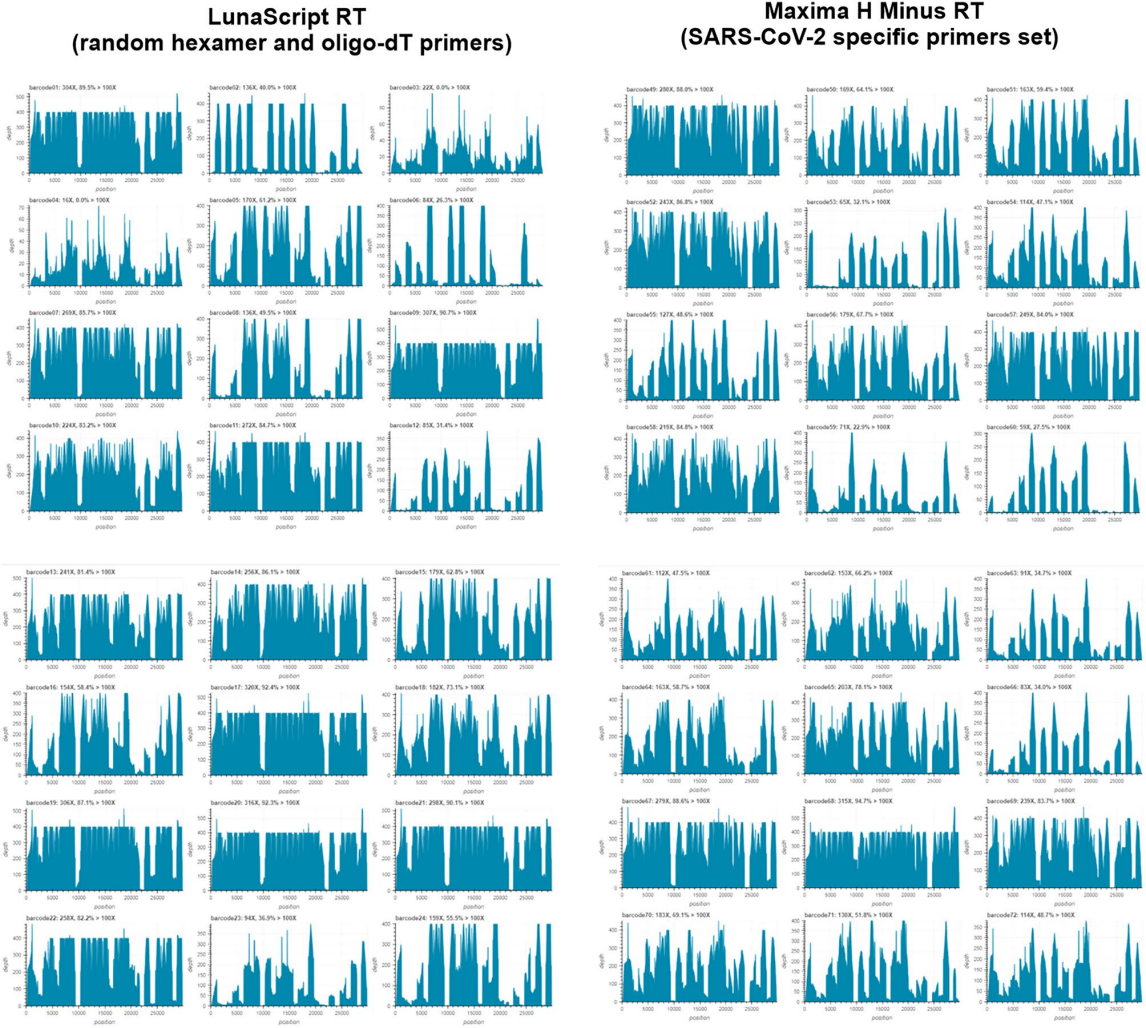
Comparative analysis for SARS-CoV-2 positive samples by ONT analysis for different cDNA synthesis protocols. Left panel, results obtained using the standard Midnight RT PCR Expansion Nanopore protocol with Midnight SARS-CoV-2 LunaScript reverse transcriptase (random hexamer and oligo-dT primers) and tiled multiplexed amplicon sets from the current ARTIC network 1.2 kb primer pool. Right panel, the same samples but with Maxima H Minus reverse transcriptase but based on a set of SARS-CoV-2 specific primers and tiled multiplexed amplicon sets from our 2.4 kb primer pool.

We tested tiled multiplexed amplicon sets from the current ARTIC network 1.2 kb primer pool, available from Midnight RT PCR Expansion (Supplementary Table S7). In the Table S9 effectively illustrates a side-by-side comparison between matching samples obtained from two sets of primers, along with their respective assigned Pango lineages. A comprehensive side-by-side analysis of matching samples obtained from two groups of primer sets and their corresponding assigned Pango lineages. By examining the columns within Table S9, we offer a compelling demonstration that, irrespective of the methodology employed, the resulting sequences consistently align with the same lineage. We compared the Nanopore protocol with our protocol for fragments in the 2.4-kb pair primer pool (Fig. 4). The same samples were used in parallel for the original protocol and our proposed protocol. All steps for the original Midnight RT PCR Expansion Nanopore protocol using the Rapid Barcoding Kit 96 were followed without change. Using our protocol, the initial steps were replaced with our reagents and primer set and primers were multiplexed into two pools (Pool A and Pool B), creating a tiled amplification of the entire SARS-CoV2 genome. After multiplex PCR using our protocol, the subsequent steps before ONT analysis followed Midnight RT PCR Expansion. Thus, the main differences between our protocol and the current ARTIC network 1.2 kb primer pool only concerned the initial steps of cDNA synthesis and multiplex PCR. Figure 5 shows the coverage of the entire SARS-CoV-2 genome for both approaches. Based on the results of ONT analysis, we observed that there are samples with equal coverage of the entire SARS-CoV-2 genome with the original protocol (Supplemental Data). The phylogenetic relatedness of the SARS-CoV-2 was reconstructed using a maximum likelihood approach by Nexclade clastidic analysis and Pangolin strain assignment based on the genomes of 341 clinical isolates. The phylogenetic tree showed 12 different types of genomic variation, including Alpha, Delta, and Omicron types (Fig. S1). In the final phylogenetic tree Fig. S1a, samples from Set 1 (Nanopore protocol) are represented by magenta-colored titles, while samples from Set 2 (our protocol) are indicated with green-colored titles. This color distinction facilitates the identification and comparison of the samples between the two sets within the tree. The phylogenetic tree reveals that samples from both Set 1 and Set 2 are clustered together, indicating the reliability of primers from Set 2. Additionally, we used Nextclade tool with merged the sequences from Set 1 (Nanopore protocol), Set 2 (our protocol), and the Wuhan reference for construction of evolutional phylogenetic tree according to Pango lineages distribution (Fig. S1b). Both groups of samples from Set1 and Set2 are clustering together in 21 K clade of omicron (Pango lineage BA.1.1). For the efficiency of primer coverage of SARS-CoV-2 variants, we analysed a total of 100 unique SARS-CoV-2 genomes obtained between 2019 and 2023 (https://www.who.int/en/activities/tracking-SARS-CoV-2-variants/). Using FastPCR software^14^, we performed an in silico analysis of the total number of complementary sites for each primer (Table S8). The primers that covered all 100 SARS-CoV-2 genomes had a hint value of 100, respectively. The border sequences of SARS-CoV-2 variants were not available for all, so the values corresponded to the number of complete sequences. As a result, most primers were fully complementary to all 100 unique variants of SARS-CoV-2. For 8 primers (A16, A27, A31, A38, A39, B4, B19, B37) mismatches in the 5'-end regions were observed, which may have slightly affected PCR efficiency. We also observed better coverage of the entire SARS-CoV-2 genome for other samples obtained with our protocol. In addition, most samples were efficiently analyzed using both approaches. The sensitivity of the ARTIC SARS-CoV-2 workflow on low-titer virus samples and RNA degradation and amplicon size limitations of the tiling PCR approach do not allow for large-scale infection monitoring over a wide range. Thus, we can confirm that in the initial stages of sample preparation for sequencing, our proposed cDNA synthesis protocol and a set of primers multiplexed into two pools can generate a tiled amplification of the entire SARS-CoV-2 genome. The protocol that is used for Maxima H Minus Reverse Transcriptase from a set of SARS-CoV-2 specific primers allows amplification greater than 5 kb. However, more sensitivity can be achieved if the same protocol is optimized for efficient amplification rather than for fragment length. In addition, we tested the efficiency of cDNA synthesis using SARS-CoV-2 specific primers but using an alternative reverse transcriptase, such as SuperScript II/III (Invitrogen). In comparison with Maxima H Minus and SuperScript II/III reverse transcriptase, the first one showed slightly higher efficiency and sensitivity in cDNA synthesis. In addition, the low cost of Maxima H Minus Reverse Transcriptase and its relatively high efficiency make this enzyme already sufficient for the application. Additionally, we used Phusion Plus DNA Polymerase (Thermo Scientific) instead of Phusion Hot Start II DNA Polymerase, which also showed excellent efficiency and sensitivity in cDNA detection in multiplex PCR.

## Discussion

Highly sensitive and efficient whole-genome sequencing of SARS-CoV-2 is critical for understanding viral transmission dynamics. For ONT, the first and most critical steps in cDNA and multiplex PCR preparation mainly determine the efficiency of SARS-CoV-2 genome coverage and the sensitivity of RNA detection in complex samples. To eliminate systematic dropouts, we provide a comprehensive multiplexed set of primers adapted for the ONT, which showed the highest genome coverage for a wide range of input RNA. In addition, our work focused on developing an efficient protocol for cDNA preparation and multiplexed PCR reactions for Pools A and B. As we used SARS-CoV-2 specific primers to synthesize the cDNA set, we achieved high sensitivity of the detection and sequencing methods for the virus genome. Primer mismatches are not a major issue affecting genome coverage, due to effective genome coverage with a new panel. Our primer scheme uses 2.4-kb amplicons for long-read sequencing platforms and has good performance with degraded or high Ct for environmental samples. The protocol proposed here allows whole-genome sequencing of SARS-CoV-2 with tiled amplicons up to 4.8 kb on low-titer virus samples and even where RNA degradation has occurred. This protocol reduces the time and costs from RNA to genome sequence compared to the Midnight multiplex PCR method for SARS-CoV-2 genome sequencing using Oxford Nanopore. The approaches and procedures proposed here may potentially be adapted for analysis of other RNA-containing viruses. The Oxford Nanopore offers multipurpose sequencing technology using the Rapid Barcoding Kit 96 for all applications and targets. The user can use existing protocols from the manufacturer or develop a protocol for the initial stages of sample preparation before barcode binding. After preparing multiplex PCR pools, samples can then be prepared for ONT sequencing using the Oxford Nanopore protocols involving the Rapid Barcoding Kit 96.

## Conclusion

We developed a new and flexible panel for ONT amplicon strategy with a comprehensive multiplexed set of primers adapted for the Oxford Nanopore rapid barcode library kit, allowing universal SARS-CoV-2 genome sequencing. A comprehensive multiplexed set of primers adapted for ONT showed the highest genome coverage for a wide range of input RNA. This primer set is designed to set up any variants of the primers pool for whole-genome sequencing of SARS-CoV-2 using single- or double-tiled amplicons from 1.2 to 4.8 kb with the Oxford Nanopore. Modifications to the library preparation protocol have improved the stage of cDNA synthesis and multiplex PCR. The high sensitivity of a cDNA synthesis protocol proposed here allows whole-genome sequencing of SARS-CoV-2 with tiled amplicons up to 4.8 kb on low-titer virus samples and even where RNA degradation has occurred. In this study, both the yield and the efficiency of cDNA synthesis with a set of SARS-CoV-2 specific primers and using Maxima H Minus Reverse Transcriptase are much higher than in the reaction with random primers and LunaScript Reverse Transcriptase. In this study, we compared different cDNA synthesis protocols and low-titer virus samples and even those in which the RNA was degraded. PCR-based target enrichment produces a high rate of on-target products with an inexpensive and simple laboratory workflow. Modifications to the library preparation protocol were successful in increasing the demultiplexing rate.

## Supplementary Information

Supplementary Information 1.

Supplementary Information 2.

## Data Availability

The datasets generated and/or analyzed during the current study are available initiative on sharing all influenza data (GISAID) (https://www.gisaid.org/hcov19-variants/) and the names of the repository/repositories and accession numbers can be found in online repositories and in the article/Supplementary Material. The sequencing data of 341 SARS-CoV-2 genomes in fastq format were deposited at National Center for Biotechnology Information Sequence Read Archive under accession number PRJNA915200 (https://www.ncbi.nlm.nih.gov/bioproject/915200).

## Abbreviations

ONT: Oxford nanopore technologies
NGS: Next-generation sequencing
TGS: Third generation sequencing
