# Supplementary material for: Universal whole-genome Oxford nanopore sequencing of SARS-CoV-2 using tiled amplicons
Source: Sci Rep. 2023 Jun 26;13:10334. doi: 10.1038/s41598-023-37588-x (PMC10293217; doi:10.1038/s41598-023-37588-x)
Supplement: Supplementary file 1 — Supplementary Information 1. [file 41598_2023_37588_MOESM1_ESM.pdf]

Supplemental Data

Results comparison with Oxford Nanopore sequencing

16/05/2022_SARS-CoV-2 Sequencing report_32 POSITIVE COVID SAMPLES (3 Sets test)

Accession date: 08/06/2023

Results generated using the wf-artic v0.3.28 Nextflow workflow provided by Oxford Nanopore Technologies.

Read Quality control

This section displays basic QC metrics indicating read data quality.

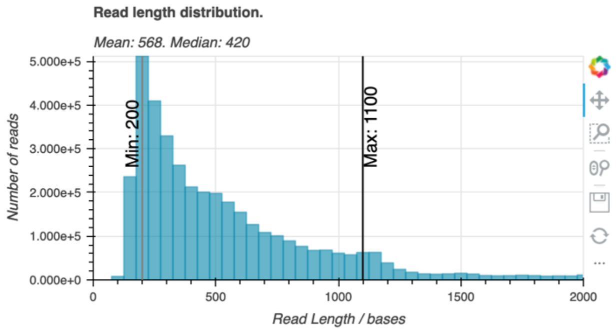

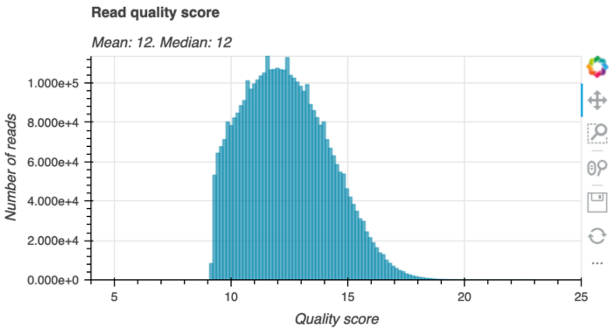

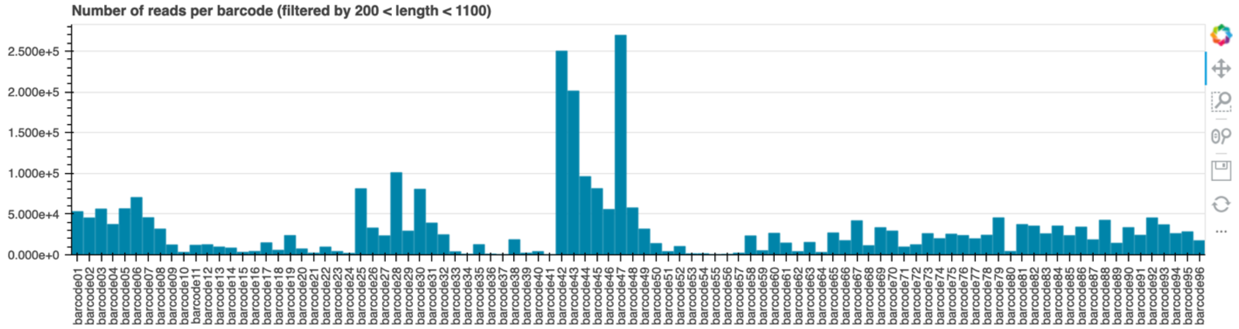

Artic Analysis status

The panel below lists samples which failed to produce results from the primary ARTIC analysis. Samples not listed here were analysed successfully, but may still contain inconclusive or invalid results. See the following sections for further indications of failed or inconclusive results.

`All samples analysed successfully`

Genome coverage

Plots below indicate depth of coverage from data used within the Artic analysis coloured by amplicon pool. Variant filtering during the ARTIC analysis mandates a minimum coverage of at least 20X at variant/genotyping loci for a call to be made.

NB: To better display all possible data, the depth axes of the plots below are not tied between plots for different samples. Care should be taken in comparing depth across samples.

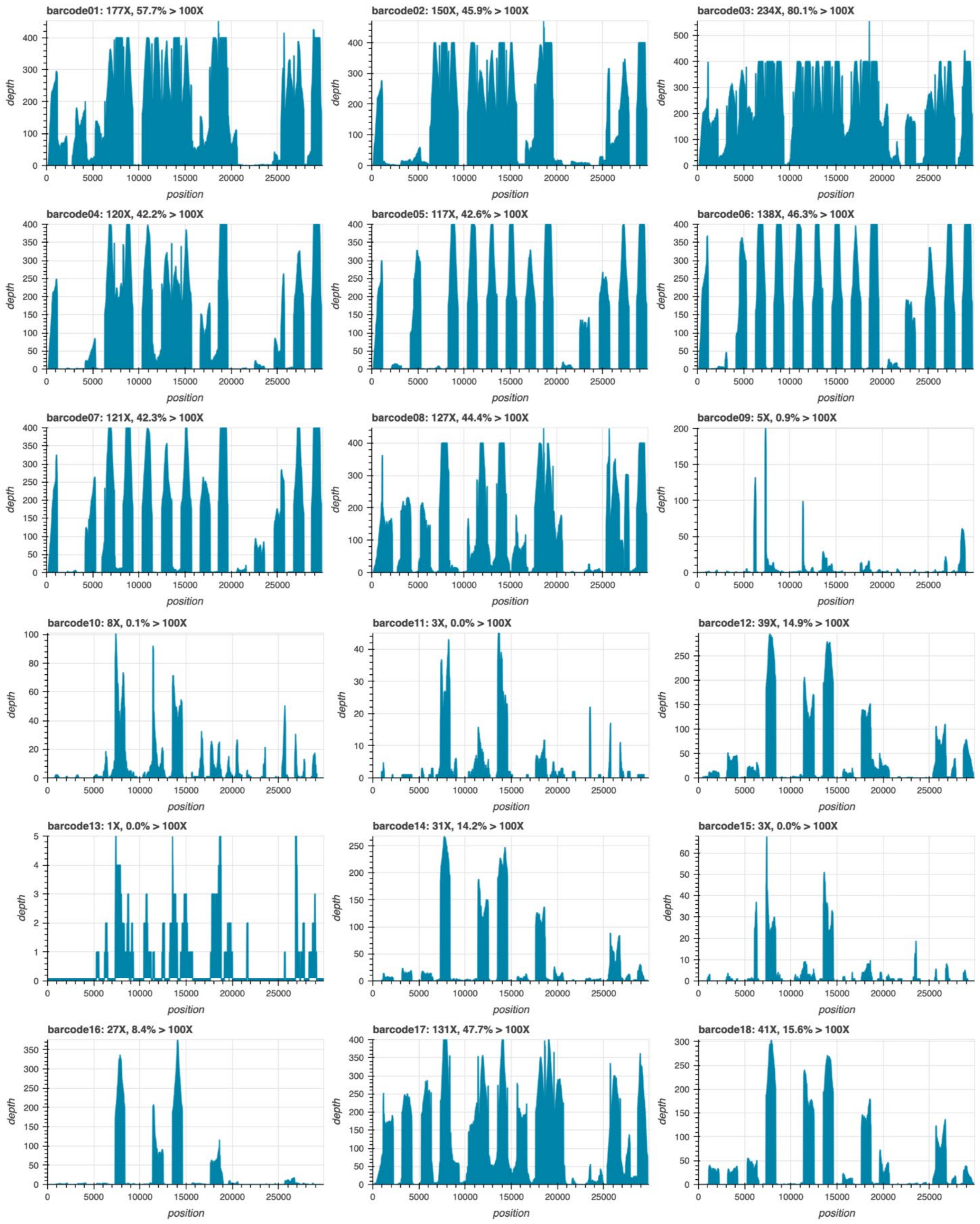
barcode01: 177X, 57.7% > 100X
barcode02: 150X, 45.9% > 100X
barcode03: 234X, 80.1% > 100X
barcode04: 120X, 42.2% > 100X
barcode05: 117X, 42.6% > 100X
barcode06: 138X, 46.3% > 100X
barcode07: 121X, 42.3% > 100X
barcode08: 127X, 44.4% > 100X
barcode09: 5X, 0.9% > 100X
barcode10: 8X, 0.1% > 100X
barcode11: 3X, 0.0% > 100X
barcode12: 39X, 14.9% > 100X
barcode13: 1X, 0.0% > 100X
barcode14: 31X, 14.2% > 100X
barcode15: 3X, 0.0% > 100X
barcode16: 27X, 8.4% > 100X
barcode17: 131X, 47.7% > 100X
barcode18: 41X, 15.6% > 100X
depth
position

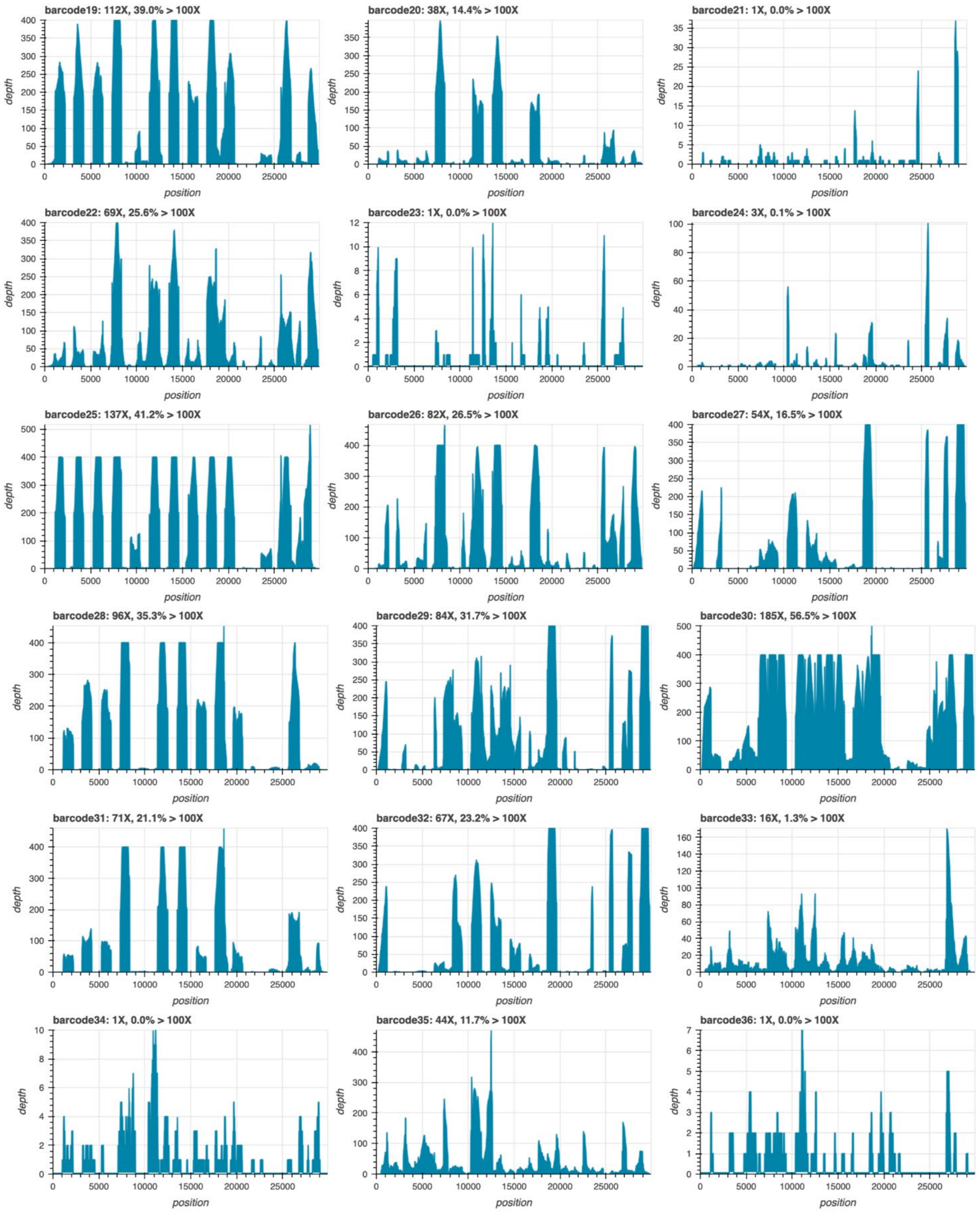
barcode19: 112X, 39.0% > 100X
barcode20: 38X, 14.4% > 100X
barcode21: 1X, 0.0% > 100X
barcode22: 69X, 25.6% > 100X
barcode23: 1X, 0.0% > 100X
barcode24: 3X, 0.1% > 100X
barcode25: 137X, 41.2% > 100X
barcode26: 82X, 26.5% > 100X
barcode27: 54X, 16.5% > 100X
barcode28: 96X, 35.3% > 100X
barcode29: 84X, 31.7% > 100X
barcode30: 185X, 56.5% > 100X
barcode31: 71X, 21.1% > 100X
barcode32: 67X, 23.2% > 100X
barcode33: 16X, 1.3% > 100X
barcode34: 1X, 0.0% > 100X
barcode35: 44X, 11.7% > 100X
barcode36: 1X, 0.0% > 100X
depth
position

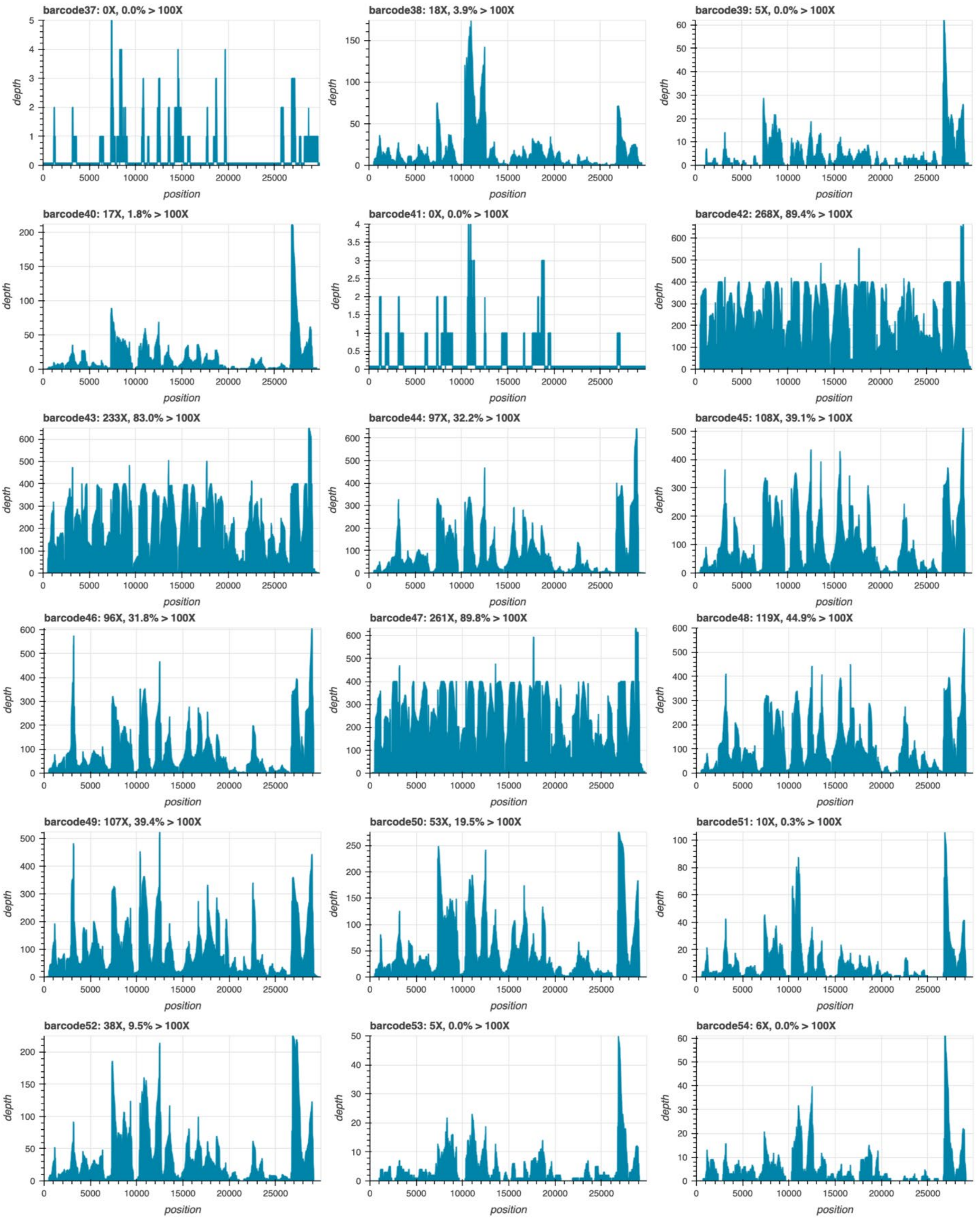
barcode37: 0X, 0.0% > 100X
barcode38: 18X, 3.9% > 100X
barcode39: 5X, 0.0% > 100X
barcode40: 17X, 1.8% > 100X
barcode41: 0X, 0.0% > 100X
barcode42: 268X, 89.4% > 100X
barcode43: 233X, 83.0% > 100X
barcode44: 97X, 32.2% > 100X
barcode45: 108X, 39.1% > 100X
barcode46: 96X, 31.8% > 100X
barcode47: 261X, 89.8% > 100X
barcode48: 119X, 44.9% > 100X
barcode49: 107X, 39.4% > 100X
barcode50: 53X, 19.5% > 100X
barcode51: 10X, 0.3% > 100X
barcode52: 38X, 9.5% > 100X
barcode53: 5X, 0.0% > 100X
barcode54: 6X, 0.0% > 100X
depth
position

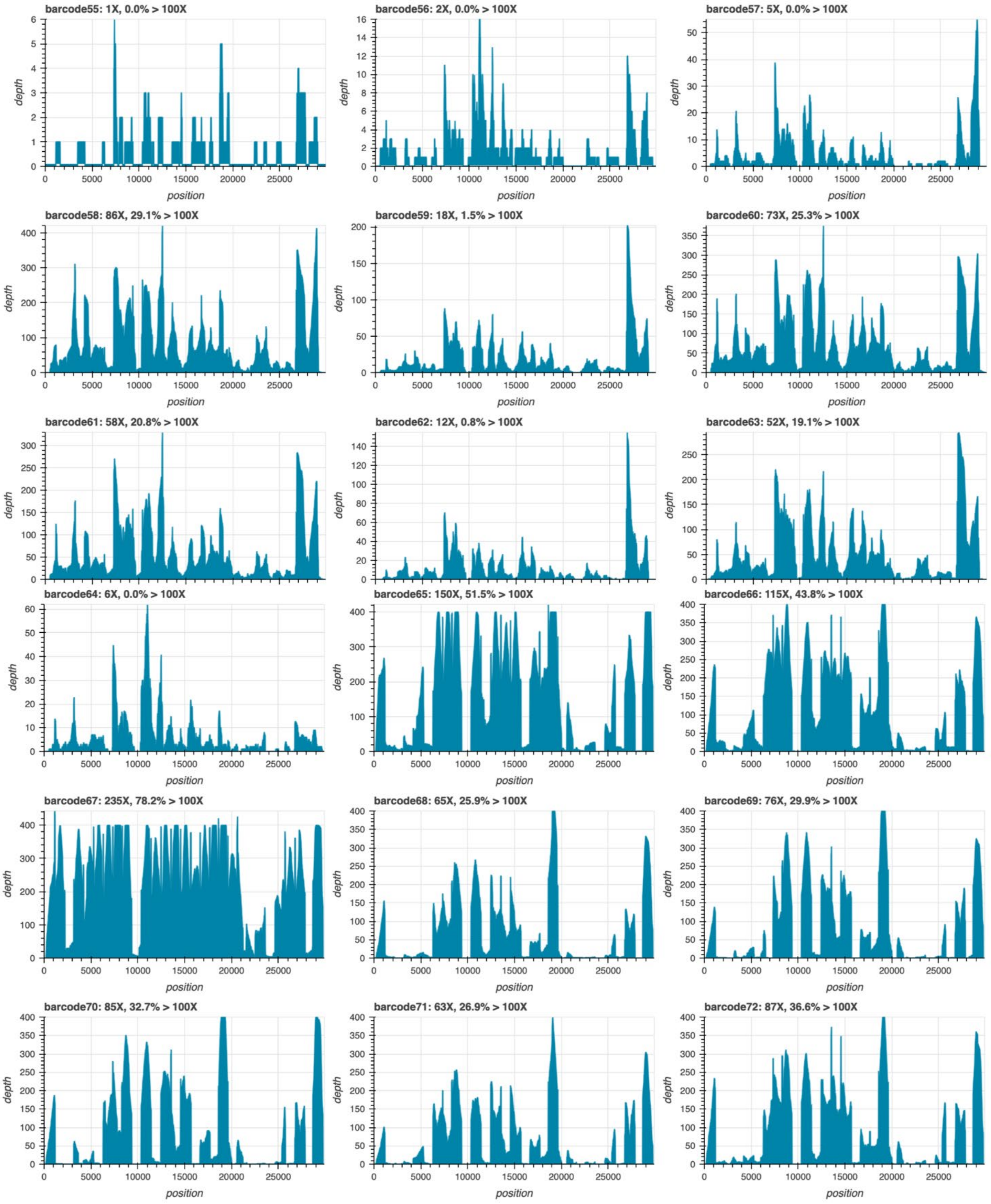
barcode55: 1X, 0.0% > 100X
barcode56: 2X, 0.0% > 100X
barcode57: 5X, 0.0% > 100X
barcode58: 86X, 29.1% > 100X
barcode59: 18X, 1.5% > 100X
barcode60: 73X, 25.3% > 100X
barcode61: 58X, 20.8% > 100X
barcode62: 12X, 0.8% > 100X
barcode63: 52X, 19.1% > 100X
barcode64: 6X, 0.0% > 100X
barcode65: 150X, 51.5% > 100X
barcode66: 115X, 43.8% > 100X
barcode67: 235X, 78.2% > 100X
barcode68: 65X, 25.9% > 100X
barcode69: 76X, 29.9% > 100X
barcode70: 85X, 32.7% > 100X
barcode71: 63X, 26.9% > 100X
barcode72: 87X, 36.6% > 100X
depth
position

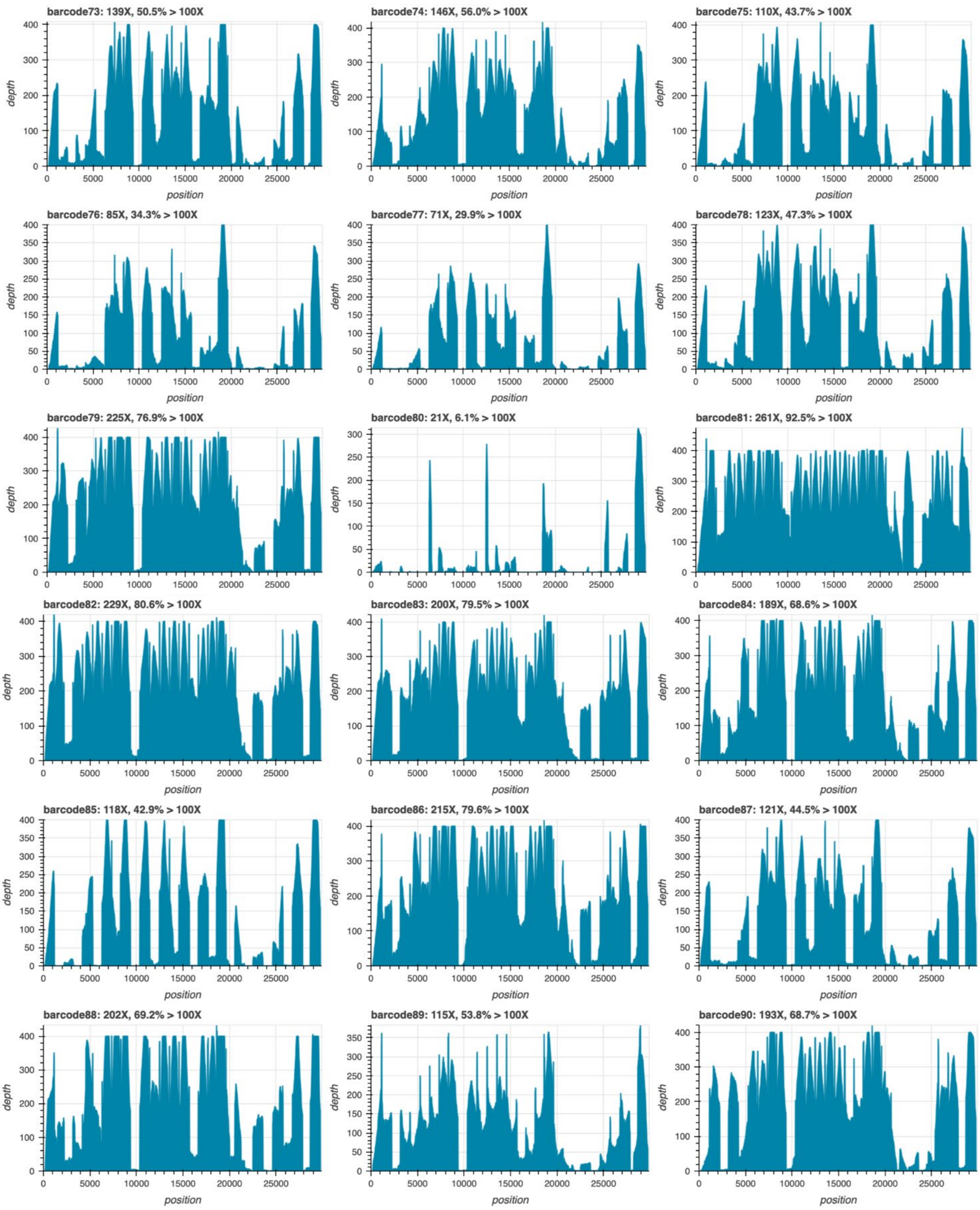
barcode73: 139X, 50.5% > 100X
barcode74: 146X, 56.0% > 100X
barcode75: 110X, 43.7% > 100X
barcode76: 85X, 34.3% > 100X
barcode77: 71X, 29.9% > 100X
barcode78: 123X, 47.3% > 100X
barcode79: 225X, 76.9% > 100X
barcode80: 21X, 6.1% > 100X
barcode81: 261X, 92.5% > 100X
barcode82: 229X, 80.6% > 100X
barcode83: 200X, 79.5% > 100X
barcode84: 189X, 68.6% > 100X
barcode85: 118X, 42.9% > 100X
barcode86: 215X, 79.6% > 100X
barcode87: 121X, 44.5% > 100X
barcode88: 202X, 69.2% > 100X
barcode89: 115X, 53.8% > 100X
barcode90: 193X, 68.7% > 100X
depth
position

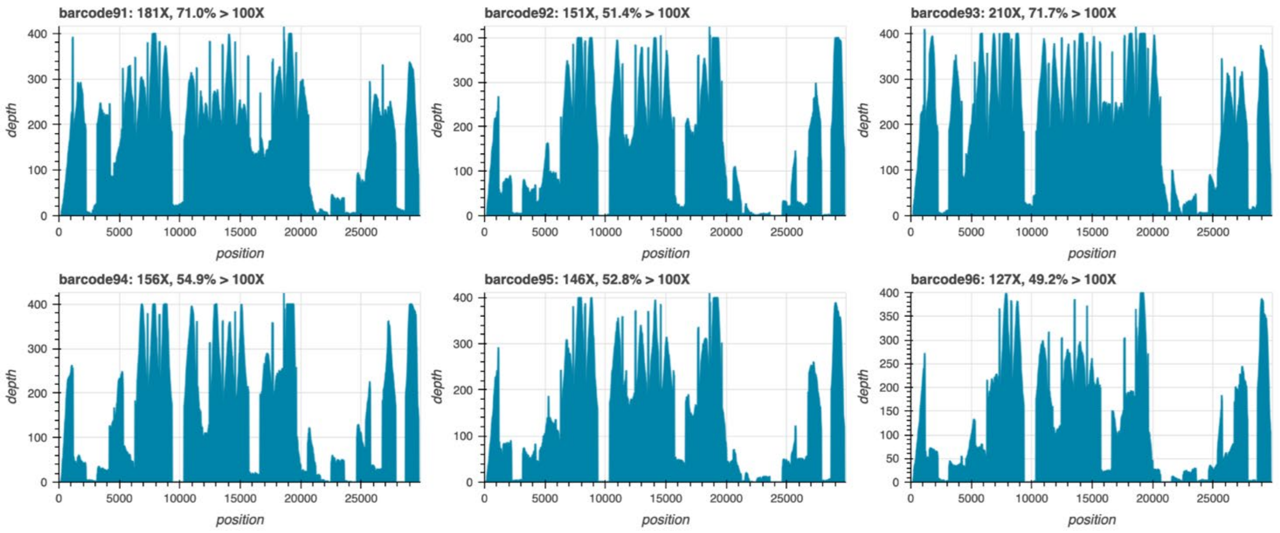

NextClade analysis

The following view is produced by the nextclade software.

i	Sequence name	QC	Clade	Pango lineage (Nextclade)	WHO name	Mut.	non-ACGTN	Ns	Cov.	Gaps
0	barcode01 MN908947.3	N M P C F S	21K	BA.1.1	Omicron	23	0	6971	76.7%	21
1	barcode02 MN908947.3	N M P C F S	21K	BA.1.1	Omicron	19	0	12641	57.7%	12
2	barcode03 MN908947.3	N M P C F S	21K	BA.1.1	Omicron	51	0	3012	89.9%	27
3	barcode04 MN908947.3	N M P C F S	21K	BA.1.1	Omicron	20	0	13091	56.2%	12
4	barcode05 MN908947.3	N M P C F S	21K	BA.1.1	Omicron	34	0	16893	43.5%	9
5	barcode06 MN908947.3	N M P C F S	21K	BA.1.1	Omicron	36	0	15440	48.4%	12
6	barcode07 MN908947.3	N M P C F S	21K	BA.1.1	Omicron	33	0	15458	48.3%	12
7	barcode08 MN908947.3	N M P C F S	21K	BA.1.1	Omicron	21	0	11513	61.5%	9
8	barcode09 MN908947.3	N M P C F S	21K	BA.1.1	Omicron	4	0	28452	4.9%	0
9	barcode10 MN908947.3	N M P C F S	21K	BA.1.1	Omicron	3	0	26384	11.8%	0
10	barcode11 MN908947.3	N M P C F S	21K	BA.1.1	Omicron	2	0	27782	7.1%	0
11	barcode12 MN908947.3	N M P C F S	21K	BA.1.1	Omicron	13	0	19674	34.2%	0
13	barcode14 MN908947.3	N M P C F S	21K	BA.1.1	Omicron	10	0	23905	20.1%	0
14	barcode15 MN908947.3	N M P C F S	21K	BA.1.1	Omicron	1	0	27571	7.8%	0
15	barcode16 MN908947.3	N M P C F S	21K	BA.1.1	Omicron	3	0	25322	15.3%	0
16	barcode17 MN908947.3	N M P C F S	21K	BA.1.1	Omicron	25	0	9236	69.1%	12
17	barcode18 MN908947.3	N M P C F S	21K	BA.1.1	Omicron	12	0	19307	35.4%	0
18	barcode19 MN908947.3	N M P C F S	21K	BA.1.1	Omicron	22	0	14676	50.9%	0
19	barcode20 MN908947.3	N M P C F S	21K	BA.1.1	Omicron	14	0	23235	22.3%	0
20	barcode21 MN908947.3	N M P C F S	21K	BA.1.1	Omicron	0	0	29587	1.1%	0
21	barcode22 MN908947.3	N M P C F S	21K	BA.1.1	Omicron	20	0	14595	51.2%	12
23	barcode24 MN908947.3	N M P C F S	21K	BA.1.1	Omicron	3	0	28533	4.6%	0
24	barcode25 MN908947.3	N M P C F S	21K	BA.1.1	Omicron	24	0	14224	52.4%	9
25	barcode26 MN908947.3	N M P C F S	21K	BA.1.1	Omicron	21	0	16819	43.8%	15
26	barcode27 MN908947.3	N M P C F S	21K	BA.1.1	Omicron	12	0	19210	35.8%	9
27	barcode28 MN908947.3	N M P C F S	recombinant	XP	recombinant	10	0	18710	37.4%	0

28	barcode29 MN908947.3	N M P C F S	21K	BA.1.1	Omicron	14	0	15622	47.8%	12
29	barcode30 MN908947.3	N M P C F S	21K	BA.1.1	Omicron	41	0	6401	78.6%	12
30	barcode31 MN908947.3	N M P C F S	21K	BA.1.1	Omicron	12	0	17880	40.2%	0
31	barcode32 MN908947.3	N M P C F S	21K	BA.1.1	Omicron	18	0	19656	34.3%	12
32	barcode33 MN908947.3	N M P C F S	21K	BA.1.1	Omicron	7	0	22771	23.9%	9
34	barcode35 MN908947.3	N M P C F S	21K	BA.1.1	Omicron	23	0	15671	47.6%	21
37	barcode38 MN908947.3	N M P C F S	21K	BA.1.1	Omicron	7	0	22675	24.2%	18
38	barcode39 MN908947.3	N M P C F S	21K	BA.1.1	Omicron	0	0	28751	3.9%	0
39	barcode40 MN908947.3	N M P C F S	21K	BA.1.1	Omicron	12	0	21844	27.0%	18
41	barcode42 MN908947.3	N M P C F S	21K	BA.1.1	Omicron	55	0	1039	96.5%	39
42	barcode43 MN908947.3	N M P C F S	21K	BA.1.1	Omicron	55	0	1466	95.1%	39
43	barcode44 MN908947.3	N M P C F S	21K	BA.1.1	Omicron	46	0	8813	70.5%	21
44	barcode45 MN908947.3	N M P C F S	21K	BA.1.1	Omicron	48	0	6938	76.8%	30
45	barcode46 MN908947.3	N M P C F S	21K	BA.1.1	Omicron	45	0	7265	75.7%	33
46	barcode47 MN908947.3	N M P C F S	21K	BA.1.1	Omicron	54	0	1122	96.2%	39
47	barcode48 MN908947.3	N M P C F S	21K	BA.1.1	Omicron	48	0	5993	80.0%	33
48	barcode49 MN908947.3	N M P C F S	21K	BA.1.1	Omicron	45	0	4297	85.6%	39
49	barcode50 MN908947.3	N M P C F S	21K	BA.1.1	Omicron	39	0	10304	65.5%	18
50	barcode51 MN908947.3	N M P C F S	21K	BA.1.1	Omicron	7	0	25657	14.2%	9
51	barcode52 MN908947.3	N M P C F S	21K	BA.1.1	Omicron	27	0	14112	52.8%	18
52	barcode53 MN908947.3	N M P C F S	21K	BA.1.1	Omicron	1	0	29134	2.6%	0
53	barcode54 MN908947.3	N M P C F S	21K	BA.1.1	Omicron	1	0	28349	5.2%	9
56	barcode57 MN908947.3	N M P C F S	21K	BA.1.1	Omicron	5	0	28267	5.5%	9
57	barcode58 MN908947.3	N M P C F S	21K	BA.1.1	Omicron	47	0	6632	77.8%	33
58	barcode59 MN908947.3	N M P C F S	21K	BA.1.1	Omicron	11	0	21511	28.1%	18
59	barcode60 MN908947.3	N M P C F S	21K	BA.1.1	Omicron	42	0	8323	72.2%	21

60	barcode61 MN908947.3	N M P C F S	21K	BA.1.1	Omicron	40	0	9381	68.6%	18
61	barcode62 MN908947.3	N M P C F S	21K	BA.1.1	Omicron	7	0	24510	18.0%	0
62	barcode63 MN908947.3	N M P C F S	21K	BA.1.1	Omicron	40	0	11371	62.0%	18
63	barcode64 MN908947.3	N M P C F S	21K	BA.1.1	Omicron	1	0	28015	6.3%	9
64	barcode65 MN908947.3	N M P C F S	21K	BA.1.1	Omicron	25	0	10401	65.2%	12
65	barcode66 MN908947.3	N M P C F S	21K	BA.1.1	Omicron	17	0	10774	64.0%	12
66	barcode67 MN908947.3	N M P C F S	21K	BA.1.1	Omicron	47	0	3071	89.7%	30
67	barcode68 MN908947.3	N M P C F S	21K	BA.1.1	Omicron	15	0	16226	45.7%	12
68	barcode69 MN908947.3	N M P C F S	21K	BA.1.1	Omicron	12	0	15533	48.1%	12
69	barcode70 MN908947.3	N M P C F S	21K	BA.1.1	Omicron	13	0	14843	50.4%	12
70	barcode71 MN908947.3	N M P C F S	21K	BA.1.1	Omicron	12	0	15228	49.1%	12
71	barcode72 MN908947.3	N M P C F S	21K	BA.1.1	Omicron	14	0	14251	52.3%	12
72	barcode73 MN908947.3	N M P C F S	21K	BA.1.1	Omicron	19	0	9257	69.0%	12
73	barcode74 MN908947.3	N M P C F S	21K	BA.1.1	Omicron	22	0	6295	78.9%	12
74	barcode75 MN908947.3	N M P C F S	21K	BA.1.1	Omicron	12	0	11976	60.0%	12
75	barcode76 MN908947.3	N M P C F S	21K	BA.1.1	Omicron	15	0	13899	53.5%	12
76	barcode77 MN908947.3	N M P C F S	21K	BA.1.1	Omicron	13	0	15112	49.5%	12
77	barcode78 MN908947.3	N M P C F S	21K	BA.1.1	Omicron	37	0	9881	67.0%	12
78	barcode79 MN908947.3	N M P C F S	21K	BA.1.1	Omicron	45	0	4179	86.0%	12
79	barcode80 MN908947.3	N M P C F S	21K	BA.1.1	Omicron	6	0	24961	16.5%	3
80	barcode81 MN908947.3	N M P C F S	21K	BA.1.1	Omicron	51	0	1362	95.4%	39
81	barcode82 MN908947.3	N M P C F S	21K	BA.1.1	Omicron	46	0	3593	88.0%	18
82	barcode83 MN908947.3	N M P C F S	21K	BA.1.1	Omicron	44	0	4815	83.9%	12
83	barcode84 MN908947.3	N M P C F S	21K	BA.1.1	Omicron	45	0	4464	85.1%	12
84	barcode85 MN908947.3	N M P C F S	21K	BA.1.1	Omicron	33	0	12582	57.9%	12
85	barcode86 MN908947.3	N M P C F S	21K	BA.1.1	Omicron	50	0	3222	89.2%	18
86	barcode87 MN908947.3	N M P C F S	21K	BA.1.1	Omicron	34	0	10915	63.5%	12
87	barcode88 MN908947.3	N M P C F S	21K	BA.1.1	Omicron	45	0	4143	86.1%	12
88	barcode89 MN908947.3	N M P C F S	21K	BA.1.1	Omicron	27	0	6220	79.2%	21
89	barcode90 MN908947.3	N M P C F S	21K	BA.1.1	Omicron	29	0	5601	81.3%	27
90	barcode91 MN908947.3	N M P C F S	21K	BA.1.1	Omicron	44	0	4472	85.0%	12
91	barcode92 MN908947.3	N M P C F S	21K	BA.1.1	Omicron	20	0	6827	77.2%	12
92	barcode93 MN908947.3	N M P C F S	21K	BA.1.1	Omicron	44	0	3775	87.4%	27
93	barcode94 MN908947.3	N M P C F S	21K	BA.1.1	Omicron	45	0	6198	79.3%	12
94	barcode95 MN908947.3	N M P C F S	21K	BA.1.1	Omicron	20	0	6580	78.0%	12
95	barcode96 MN908947.3	N M P C F S	21K	BA.1.17.2	Omicron	40	0	6297	78.9%	12

Lineage

The table below reports the lineage of each sample as calculated by pangolin.

taxon	lineage	Scorpio_call	version	Qc_status
**barcode01 MN908947.3**	BA.1.1	Omicron (BA.1-like)	PUSHER-v1.18.1.1	pass
**barcode02 MN908947.3**	BA.1.1	Omicron (BA.1-like)	PUSHER-v1.18.1.1	pass
**barcode03 MN908947.3**	BA.1.1	Omicron (BA.1-like)	PUSHER-v1.18.1.1	pass
**barcode04 MN908947.3**	BA.1.1	Omicron (BA.1-like)	PUSHER-v1.18.1.1	pass
**barcode05 MN908947.3**	BA.1.1	Omicron (BA.1-like)	PUSHER-v1.18.1.1	pass
**barcode06 MN908947.3**	BA.1.1	Omicron (BA.1-like)	PUSHER-v1.18.1.1	pass
**barcode07 MN908947.3**	BA.1.1	Omicron (BA.1-like)	PUSHER-v1.18.1.1	pass
**barcode08 MN908947.3**	BA.1.1	Omicron (BA.1-like)	PUSHER-v1.18.1.1	pass
**barcode09 MN908947.3**	BA.1.1	Omicron (BA.1-like)	PUSHER-v1.18.1.1	pass
**barcode10 MN908947.3**	BA.1.1	Omicron (BA.1-like)	PUSHER-v1.18.1.1	pass
**barcode11 MN908947.3**	BA.1.1	Omicron (BA.1-like)	PUSHER-v1.18.1.1	pass
**barcode12 MN908947.3**	BA.1.1	Omicron (BA.1-like)	PUSHER-v1.18.1.1	pass
**barcode13 MN908947.3**	Unassigned	NaN	PUSHER-v1.18.1.1	fail
**barcode14 MN908947.3**	BA.1.1	Omicron (BA.1-like)	PUSHER-v1.18.1.1	pass
**barcode15 MN908947.3**	BA.1.1	Omicron (BA.1-like)	PUSHER-v1.18.1.1	pass
**barcode16 MN908947.3**	BA.1.1	Omicron (BA.1-like)	PUSHER-v1.18.1.1	pass
**barcode17 MN908947.3**	BA.1.1	Omicron (BA.1-like)	PUSHER-v1.18.1.1	pass
**barcode18 MN908947.3**	BA.1.1	Omicron (BA.1-like)	PUSHER-v1.18.1.1	pass
**barcode19 MN908947.3**	BA.1.1	Omicron (BA.1-like)	PUSHER-v1.18.1.1	pass
**barcode20 MN908947.3**	BA.1.1	Omicron (BA.1-like)	PUSHER-v1.18.1.1	pass
**barcode21 MN908947.3**	BA.1.1	Omicron (BA.1-like)	PUSHER-v1.18.1.1	pass
**barcode22 MN908947.3**	BA.1.1	Omicron (BA.1-like)	PUSHER-v1.18.1.1	pass
**barcode23 MN908947.3**	Unassigned	NaN	PUSHER-v1.18.1.1	fail
**barcode24 MN908947.3**	BA.1.1	Omicron (BA.1-like)	PUSHER-v1.18.1.1	pass
**barcode25 MN908947.3**	BA.1.1	Omicron (BA.1-like)	PUSHER-v1.18.1.1	pass
**barcode26 MN908947.3**	BA.1.1	Omicron (BA.1-like)	PUSHER-v1.18.1.1	pass
**barcode27 MN908947.3**	BA.1.1	Omicron (BA.1-like)	PUSHER-v1.18.1.1	pass
**barcode28 MN908947.3**	XP	Recombinant	PUSHER-v1.18.1.1	pass
**barcode29 MN908947.3**	BA.1.1	Omicron (BA.1-like)	PUSHER-v1.18.1.1	pass
**barcode30 MN908947.3**	BA.1.1	Omicron (BA.1-like)	PUSHER-v1.18.1.1	pass
**barcode31 MN908947.3**	BA.1.1	Omicron (BA.1-like)	PUSHER-v1.18.1.1	pass
**barcode32 MN908947.3**	BA.1.1	Omicron (BA.1-like)	PUSHER-v1.18.1.1	pass
**barcode33 MN908947.3**	BA.1.1	Omicron (BA.1-like)	PUSHER-v1.18.1.1	pass
**barcode34 MN908947.3**	Unassigned	NaN	PUSHER-v1.18.1.1	fail
**barcode35 MN908947.3**	BA.1.1	Omicron (BA.1-like)	PUSHER-v1.18.1.1	pass
**barcode36 MN908947.3**	Unassigned	NaN	PUSHER-v1.18.1.1	fail
**barcode37 MN908947.3**	Unassigned	NaN	PUSHER-v1.18.1.1	fail
**barcode38 MN908947.3**	BA.1.1	Omicron (BA.1-like)	PUSHER-v1.18.1.1	pass
**barcode39 MN908947.3**	BA.1.1	Omicron (BA.1-like)	PUSHER-v1.18.1.1	pass
**barcode40 MN908947.3**	BA.1.1	Omicron (BA.1-like)	PUSHER-v1.18.1.1	pass
**barcode41 MN908947.3**	Unassigned	NaN	PUSHER-v1.18.1.1	fail
**barcode42 MN908947.3**	BA.1.1	Omicron (BA.1-like)	PUSHER-v1.18.1.1	pass
**barcode43 MN908947.3**	BA.1.1	Omicron (BA.1-like)	PUSHER-v1.18.1.1	pass
**barcode44 MN908947.3**	BA.1.1	Omicron (BA.1-like)	PUSHER-v1.18.1.1	pass
**barcode45 MN908947.3**	BA.1.1	Omicron (BA.1-like)	PUSHER-v1.18.1.1	pass
**barcode46 MN908947.3**	BA.1.1	Omicron (BA.1-like)	PUSHER-v1.18.1.1	pass
**barcode47 MN908947.3**	BA.1.1	Omicron (BA.1-like)	PUSHER-v1.18.1.1	pass
**barcode48 MN908947.3**	BA.1.1	Omicron (BA.1-like)	PUSHER-v1.18.1.1	pass
**barcode49 MN908947.3**	BA.1.1	Omicron (BA.1-like)	PUSHER-v1.18.1.1	pass
**barcode50 MN908947.3**	BA.1.1	Omicron (BA.1-like)	PUSHER-v1.18.1.1	pass
**barcode51 MN908947.3**	BA.1.1	Omicron (BA.1-like)	PUSHER-v1.18.1.1	pass
**barcode52 MN908947.3**	BA.1.1	Omicron (BA.1-like)	PUSHER-v1.18.1.1	pass
**barcode53 MN908947.3**	BA.1.1	Omicron (BA.1-like)	PUSHER-v1.18.1.1	pass
**barcode54 MN908947.3**	BA.1.1	Omicron (BA.1-like)	PUSHER-v1.18.1.1	pass
**barcode55 MN908947.3**	Unassigned	NaN	PUSHER-v1.18.1.1	fail
**barcode56 MN908947.3**	Unassigned	NaN	PUSHER-v1.18.1.1	fail
**barcode57 MN908947.3**	BA.1.1	Omicron (BA.1-like)	PUSHER-v1.18.1.1	pass
**barcode58 MN908947.3**	BA.1.1	Omicron (BA.1-like)	PUSHER-v1.18.1.1	pass
**barcode59 MN908947.3**	BA.1.1	Omicron (BA.1-like)	PUSHER-v1.18.1.1	pass
**barcode60 MN908947.3**	BA.1.1	Omicron (BA.1-like)	PUSHER-v1.18.1.1	pass
**barcode61 MN908947.3**	BA.1.1	Omicron (BA.1-like)	PUSHER-v1.18.1.1	pass
**barcode62 MN908947.3**	BA.1.1	Omicron (BA.1-like)	PUSHER-v1.18.1.1	pass
**barcode63 MN908947.3**	BA.1.1	Omicron (BA.1-like)	PUSHER-v1.18.1.1	pass
**barcode64 MN908947.3**	BA.1.1	Omicron (BA.1-like)	PUSHER-v1.18.1.1	pass
**barcode65 MN908947.3**	BA.1.1	Omicron (BA.1-like)	PUSHER-v1.18.1.1	pass
**barcode66 MN908947.3**	BA.1.1	Omicron (BA.1-like)	PUSHER-v1.18.1.1	pass
**barcode67 MN908947.3**	BA.1.1	Omicron (BA.1-like)	PUSHER-v1.18.1.1	pass

**barcode68 MN908947.3**	BA.1.1	Omicron (BA.1-like)	PUSHER-v1.18.1.1	pass
**barcode69 MN908947.3**	BA.1.1	Omicron (BA.1-like)	PUSHER-v1.18.1.1	pass
**barcode70 MN908947.3**	BA.1.1	Omicron (BA.1-like)	PUSHER-v1.18.1.1	pass
**barcode71 MN908947.3**	BA.1.1	Omicron (BA.1-like)	PUSHER-v1.18.1.1	pass
**barcode72 MN908947.3**	BA.1.1	Omicron (BA.1-like)	PUSHER-v1.18.1.1	pass
**barcode73 MN908947.3**	BA.1.1	Omicron (BA.1-like)	PUSHER-v1.18.1.1	pass
**barcode74 MN908947.3**	BA.1.1	Omicron (BA.1-like)	PUSHER-v1.18.1.1	pass
**barcode75 MN908947.3**	BA.1.1	Omicron (BA.1-like)	PUSHER-v1.18.1.1	pass
**barcode76 MN908947.3**	BA.1.1	Omicron (BA.1-like)	PUSHER-v1.18.1.1	pass
**barcode77 MN908947.3**	BA.1.1	Omicron (BA.1-like)	PUSHER-v1.18.1.1	pass
**barcode78 MN908947.3**	BA.1.1	Omicron (BA.1-like)	PUSHER-v1.18.1.1	pass
**barcode79 MN908947.3**	BA.1.1	Omicron (BA.1-like)	PUSHER-v1.18.1.1	pass
**barcode80 MN908947.3**	BA.1.1	Omicron (BA.1-like)	PUSHER-v1.18.1.1	pass
**barcode81 MN908947.3**	BA.1.1	Omicron (BA.1-like)	PUSHER-v1.18.1.1	pass
**barcode82 MN908947.3**	BA.1.1	Omicron (BA.1-like)	PUSHER-v1.18.1.1	pass
**barcode83 MN908947.3**	BA.1.1	Omicron (BA.1-like)	PUSHER-v1.18.1.1	pass
**barcode84 MN908947.3**	BA.1.1	Omicron (BA.1-like)	PUSHER-v1.18.1.1	pass
**barcode85 MN908947.3**	BA.1.1	Omicron (BA.1-like)	PUSHER-v1.18.1.1	pass
**barcode86 MN908947.3**	BA.1.1	Omicron (BA.1-like)	PUSHER-v1.18.1.1	pass
**barcode87 MN908947.3**	BA.1.1	Omicron (BA.1-like)	PUSHER-v1.18.1.1	pass
**barcode88 MN908947.3**	BA.1.1	Omicron (BA.1-like)	PUSHER-v1.18.1.1	pass
**barcode89 MN908947.3**	BA.1.1	Omicron (BA.1-like)	PUSHER-v1.18.1.1	pass
**barcode90 MN908947.3**	BA.1.1	Omicron (BA.1-like)	PUSHER-v1.18.1.1	pass
**barcode91 MN908947.3**	BA.1.1	Omicron (BA.1-like)	PUSHER-v1.18.1.1	pass
**barcode92 MN908947.3**	BA.1.1	Omicron (BA.1-like)	PUSHER-v1.18.1.1	pass
**barcode93 MN908947.3**	BA.1.1	Omicron (BA.1-like)	PUSHER-v1.18.1.1	pass
**barcode94 MN908947.3**	BA.1.1	Omicron (BA.1-like)	PUSHER-v1.18.1.1	pass
**barcode95 MN908947.3**	BA.1.1	Omicron (BA.1-like)	PUSHER-v1.18.1.1	pass
**barcode96 MN908947.3**	BA.1.17.2	Omicron (BA.1-like)	PUSHER-v1.18.1.1	pass

Conclusion

After analysis of all 96 samples, 88 (91.7%) of them passed QC and further analysis with lineage identification, and 8 samples (8.3%) failed QC.

From 32 samples of each Set1, Set2, Set3:

- 30 samples passed – Set 1 (2 failed, ONT primers)
- 26 samples passed – Set 2 (6 failed, Custom primers)
- 32 samples passed – Set 3 (0 failed, mix ONT primers + Custom primers)

Among 88 passed almost all samples revealed as Omicron strain with distinguished lineages:

- BA.1.1 (86 samples, 97.8%)
- BA.1.17.2 (1 sample, 1.1%)
- XP Recombinant (1 sample, 1.1%)
